# Single-domain antibody–based noninvasive in vivo imaging of α-synuclein or tau pathology

**DOI:** 10.1126/sciadv.adf3775

**Published:** 2023-05-10

**Authors:** Yixiang Jiang, Yan Lin, Senthilkumar Krishnaswamy, Ruimin Pan, Qian Wu, Leslie A. Sandusky-Beltran, Mengyu Liu, Min-Hao Kuo, Xiang-Peng Kong, Erin E. Congdon, Einar M. Sigurdsson

**Affiliations:** ^1^ Department of Neuroscience and Physiology, Neuroscience Institute, New York University Grossman School of Medicine, 435 East 30th Street, New York, NY 10016, USA.; ^2^ Department of Biochemistry and Molecular Pharmacology, New York University Grossman School of Medicine, 550 First Avenue, New York, NY 10016, USA.; ^3^ Department of Biochemistry and Molecular Biology, Michigan State University, 603 Wilson Road, East Lansing, MI 48824, USA.; ^4^ Department of Psychiatry, New York University Grossman School of Medicine, 435 East 30th Street, New York, NY 10016, USA.

## Abstract

Intracellular deposition of α-synuclein and tau are hallmarks of synucleinopathies and tauopathies, respectively. Recently, several dye-based imaging probes with selectivity for tau aggregates have been developed, but suitable imaging biomarkers for synucleinopathies are still unavailable. Detection of both of these aggregates early in the disease process may allow for prophylactic therapies before functional impairments have manifested, highlighting the importance of developing specific imaging probes for these lesions. In contrast to the β sheet dyes, single-domain antibodies, found in camelids and a few other species, are highly specific, and their small size allows better brain entry and distribution than whole antibodies. Here, we have developed such imaging ligands via phage display libraries derived from llamas immunized with α-synuclein and tau preparations, respectively. These probes allow noninvasive and specific in vivo imaging of α-synuclein versus tau pathology in mice, with the brain signal correlating strongly with lesion burden. These small antibody derivatives have great potential for in vivo diagnosis of these diseases.

## Teaser

The potential of small antibody fragment-based diagnostic in vivo imaging of α-synuclein or tau pathology for earlier diagnosis.

## INTRODUCTION

Synucleinopathies and tauopathies are characterized by progressive deposition of α-synuclein (α-syn) and tau proteins, respectively, and typically within neurons (*1*–*3*). Familial forms of these diseases have been linked to mutations within the proteins or to duplication or triplication of the synuclein gene, indicating a prominent role of these proteins in the etiology and pathogenesis of these diseases. Consequently, being able to image these aggregates in intact live subjects would allow early and accurate diagnosis that may aid in clinical trials on drugs targeting these lesions and eventually for prophylactic therapies. Notably, the development of these deposits is thought to have initiated years and even decades before functional impairments become evident (*4*–*6*). Therefore, it would be helpful to be able to detect these lesions in their earliest stage to facilitate their therapeutic clearance before irreversible damage. Likewise, specific detection of these different proteins is important because it is not uncommon for individuals to have features of both of these different pathologies (*7*). Hence, it can be difficult to predict who is a candidate for therapies targeting these different proteins, which would be greatly facilitated by the development of specific imaging agents. Currently, there are no approved therapies that affect the progression of these diseases, but several approaches are in clinical trials (*8*, *9*).

Progress has been made in developing fluid biomarkers for these diseases, but those are mostly if not solely used experimentally. It has been well established that levels of tau and some of its phospho forms are increased in the cerebrospinal fluid (CSF) in patients with Alzheimer’s but not in the primary tauopathies (*10*). Likewise, recent advances in blood-based biomarker detection have given similar results as seen previously in the CSF (*10*). Findings are more inconsistent for α-syn, but various improvements are in development (*11*). On the imaging front, small-molecule probes have been developed to detect by positron emission tomography (PET) the defining β sheet amyloid aggregates in Alzheimer’s disease (AD) that are composed of β-amyloid (Aβ) peptide and tau protein. Although not specific, these dye-based molecules can sufficiently distinguish between the two. A few of these Aβ and tau PET tracers are already in clinical use with second-generation β sheet binders in clinical development that can detect at least some of the non-Alzheimer’s tau lesions (*12*–*14*). However, since their general target is β sheet structures, they will always have some affinity for unrelated proteins and peptides, such as various amyloids that may deposit in the brain. The β sheet dyes that have been reported to detect α-syn aggregates bind strongly to Aβ and other β sheet structures and have not been deemed suitable for clinical use for α-syn imaging (*15*–*17*).

Antibody-derived imaging compounds should have much higher specificity for tau than β sheet binders, and most tau antibodies recognize tau lesions in all tauopathies. However, whole antibodies (150 kDa) do not get into the brain in sufficient quantities for PET detection of tau aggregates, and their half-life of several weeks is unnecessarily long for an imaging compound. We previously reported promising results using a single-chain variable fragment (scFv, 30 kDa) of a tau-targeting antibody as a diagnostic imaging agent (*18*). More suitable are single-domain antibodies (sdAbs, 15 kDa) that typically are more soluble than scFvs and have a higher affinity for their target. In addition, because of their smaller size, sdAbs get into the brain in larger quantities, are more likely to bind to previously unidentified cryptic epitopes and have a shorter and thereby appropriate half-life as imaging probes.

**Fig. 1.**
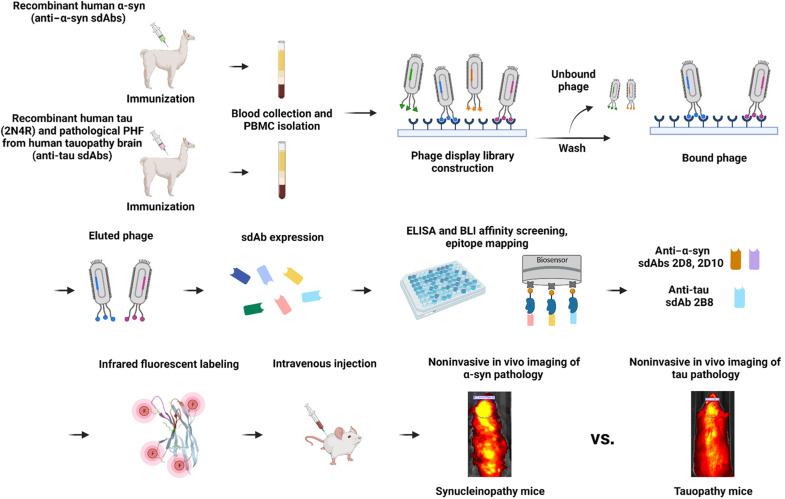
An overview of the development of the anti–α-syn (2D8, 2D10) versus anti-tau (2B8) sdAb imaging probes for noninvasive detection of synucleinopathy versus tauopathy in living animals. Initially, llamas were immunized with rec α-syn (anti–α-syn sdAbs) versus rec tau (2N4R) and PHF-enriched tau from human tauopathy brain (anti-tau sdAbs), followed by generation of phage display libraries from high titer bleeds and then selection of promising sdAbs for diagnostic imaging of α-syn versus tau pathology. PBMC, peripheral blood mononuclear cells; ELISA, enzyme-linked immunosorbent assay; BLI, biolayer interferometry.

To develop these sdAbs, we immunized different llamas with full-length recombinant human α-syn (rec α-syn) versus the longest isoform of recombinant human tau (rec tau) and enriched pathological tau isolated from a human tauopathy brain. Phage display libraries of sdAbs panned against the recombinant proteins in the solid and solution phase were subsequently generated from peripheral blood mononuclear cells (PBMCs) of the llama. Following their characterization in various assays, a few sdAbs were selected for in vivo imaging to determine their diagnostic potential (Fig. 1). We here report that their brain signal in intact synucleinopathy versus tauopathy mice correlated strongly with α-syn and tau burden in their brains, and the sdAbs could be detected inside neurons bound specifically to pathological α-syn versus tau within the endosomal-lysosomal system. These findings support their further development as specific imaging agents and may lead to clinical diagnostic markers for various diseases characterized by depositions of these aggregates alone, together, and with other aggregates. These probes may also allow monitoring the efficacy of therapies targeting these aggregates.

## RESULTS

### α-Syn versus tau immunization and sdAb characterization

Llamas were immunized with (i) rec α-syn (140 amino acids) (for the anti–α-syn sdAbs) as per protocol in table S1 (see Materials and Methods) or (ii) full-length recombinant longest isoform (2N4R) of rec tau for the first five immunizations and then with enriched paired helical filament (PHF) tau isolated from human tauopathy brain for the last two immunizations (for the anti-tau sdAbs) as per protocol in table S2 (see Materials and Methods).

#### Anti–α-syn sdAbs

Some autoantibodies that recognized the immunogen were detected in the preimmune bleed, and the antibody titer increased in subsequent bleeds, peaking in bleeds 5 and 6 (fig. S1A). The sera from these bleeds were also reacted on a dot blot with a low-speed supernatant (LSS) of brain homogenates from two subjects who had been diagnosed as having Lewy body dementia (LBD), which is characterized by extensive α-syn brain deposition. The strongest reaction was detected in bleed 4 (fig. S1B). Generally speaking, with additional immunizations, antibody diversity is known to decrease and the affinity of individual clones is known to increase. With this in mind, PBMCs were isolated from bleeds 4 and 5, and phage display sdAb libraries were developed from a mixture of them. Numerous clones bound strongly to the rec α-syn immunogen in its solution phase or solid phase (fig. S2, A and B). Subsequent sequencing revealed that 58 (43 solid phase and 15 solution phase) of those had unique antigen binding regions [complementarity-determining regions (CDRs)].

#### Anti-tau sdAbs

Some autoantibodies that recognized the rec tau immunogen were detected in the preimmune bleed, and the antibody titer increased substantially in the second bleed and remained at that level throughout subsequent bleeds (fig. S1C). The sera from these bleeds were also reacted on a dot blot with PHF-enriched tau from two human tauopathy subjects. The strongest reaction was detected in bleed 4 (fig. S1D). PBMCs were isolated from bleed 4 because of its strongest reaction with the tauopathy brains and from bleed 6 to incorporate clones that may have been generated against the PHF immunogen. A phage display sdAb library was developed from a mixture of them. Numerous clones were identified that bound strongly to the rec tau immunogen in its solution phase or solid phase (fig. S2, C and D). Some of these also bound strongly to the PHF immunogen (fig. S3). In the solution phase, the clones could be separated into two groups, one with affinities for both PHF and rec tau and another one with affinity only for PHF (fig. S3, A and B). In the solid phase, more clone diversity was detected with several clones showing varying affinity toward the two different immunogens (fig. S3, C and D). Subsequent sequencing revealed that 55 (42 solid phase and 13 solution phase) of those clones had unique antigen binding regions (CDRs).

Preliminary screening of the bacterial culture supernatants expressing the anti–α-syn and anti-tau sdAb clones examining their enzyme-linked immunosorbent assay (ELISA) binding to different α-syn and tau preparations revealed diverse binding profiles, and selected representative clones were then scaled up, purified, and screened for their ability to stain α-syn and tau lesions on brain sections. 

#### Anti–α-syn sdAbs

**Fig. 2.**
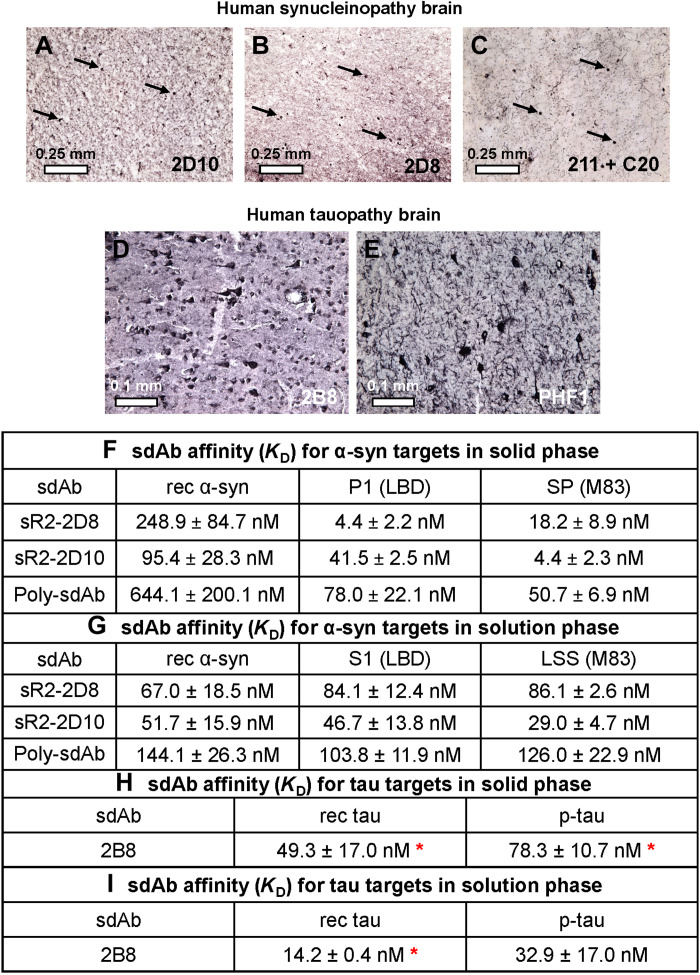
Immunohistochemistry and affinity of anti–α-syn and anti-tau sdAbs. (**A** to **E**) SdAb binding to synucleinopathy (A to C) versus tauopathy (D and E) brains. Monoclonal sdAbs-2D10 and -2D8 stain numerous Lewy bodies (α-syn inclusions, arrows) in a cortical section of a human LBD brain. (C) For comparison, staining of an adjacent section of the same brain with a mixture of anti–α-syn monoclonal antibody 211 and polyclonal antibody C20 reveals a similar number of Lewy bodies as with the sdAbs, in addition to Lewy neurites that are not as readily visible with the sdAbs. (D) Monoclonal sdAb-2B8 stains numerous tangles and pretangles in a human tauopathy brain (mixed AD/Pick’s disease, the same brain as used for PHF extraction for immunizations 6 and 7). (E) For comparison, PHF1 staining of a phospho-tau epitope of an adjacent section of the same brain reveals mostly mature tangles, dystrophic neurites, and possibly glial cells. sdAb affinity for α-syn (F and G) versus tau target (H and I). (**F**) 2D8 and 2D10 affinity for rec α-syn, insoluble pellet fraction (P1) from LBD brain, and insoluble SP fraction from Tg-M83 mouse brain, in the solid phase measured by BLI assays. (**G**) 2D8 and 2D10 affinity for rec α-syn, soluble fraction (S1) from LBD brain, and LSS soluble fraction from tg-M83 mouse brain in the solution phase in BLI assays. (**H**) 2B8 affinity for rec 2N4R tau and rec 1N4R p-tau in the solid phase. (**I**) Affinity of 2B8 toward rec tau and p-tau in the solution phase. See tables S7 and S8 for the association (*k*_a_) and dissociation (*k*_d_) values. *Some data in (H) and (I), without the fitting curves, have been previously published as part of a larger figure when examining the efficacies of 2B8 and another anti-tau sdAb (*47*).

sdAbs 2D8 and 2D10 stood out for their ability to bind very strongly to Lewy bodies (Fig. 2, A and B), whereas staining of an adjacent brain section with anti–α-syn monoclonal antibody syn 211 and polyclonal antibody C20 (Fig. 2C) revealed a similar number of Lewy bodies as with the sdAbs. In addition, the positive control antibodies bound well to Lewy neurites that were not as readily visible with the sdAbs. To further test the specificity of the sdAbs, we performed a dot blot assay using both human- and mouse-derived brain samples.

For the human samples, sdAbs 2D10 and 2D8 bound strongly to the LBD brain tissue but had limited reactivity to the control brain homogenate or the PHF-enriched tau fraction (fig. S4, A and B). Likewise, positive controls anti–α-syn polyclonal antibody PA5-13401 and anti–α-syn monoclonal phospho-serine 129 (p-S129) antibody bound strongly to LBD tissue and weakly to control tissue (fig. S4C). Some reactivity of PA5-13401 to PHF likely reflects some α-syn aggregates that enrich with the PHF fraction. The p-S129 antibody only reacted strongly with one of the LBD brains, which presumably reflects more α-syn pathology in that brain (fig. S4D), as supported by stronger binding of 2D10, 2D8, and PA5-13401 to that LBD brain compared to the other LBD brain (fig. S4, A to C). This is consistent with the dot blots from fig. S1, where the reactivity of the bleeds was stronger in the same sample.

For the mouse samples, anti–α-syn sdAbs 2D10, 2D8, and antibody PA5-13401 bound strongly to the A53T α-syn transgenic (tg) M83 brain homogenates, with limited binding to wild-type (WT) control or P301L tau tg JNPL3 brain homogenates (fig. S5, A to C). Anti–α-syn p-S129 antibody had limited reactivity for all the mouse samples (fig. S5D). These results indicate the specificity of sdAbs 2D10 and 2D8 for synucleinopathy compared to tauopathy or other nonspecific targets in control tissue. In addition, these sdAbs appear to be more sensitive to detect α-syn lesions than p-S129 antibody.

Subsequently, we determined the binding affinities of sdAbs 2D8 and 2D10 to different α-syn preparations in solid and solution phases by a biolayer interferometry (BLI) assay (Fig. 2, F and G, and figs. S6 and S7). Both sdAbs were derived from the solid-phase panning, and to be useful as imaging agents, high affinities of sdAbs for insoluble α-syn in a solid phase are likely to be more important than their affinities for various α-syn preparations in the solution phase.

2D8 had the highest affinity for the insoluble fraction from a human LBD brain (*K*_D_ = 4.4 ± 2.2 nM), followed by the insoluble fraction from a tg M83 mouse brain (*K*_D_ = 18.2 ± 8.9 nM), and lowest affinity for rec α-syn (*K*_D_ = 248.9 ± 84.7 nM) (Fig. 2F and fig. S6). 2D10, on the other hand, had the highest affinity for the insoluble fraction from tg M83 mouse brain (*K*_D_ = 4.4 ± 2.3 nM), followed by the insoluble fraction from a human LBD brain (*K*_D_ = 41.5 ± 2.5 nM), and lowest affinity for rec α-syn (*K*_D_ = 95.4 ± 28.3 nM) (Fig. 2F and fig. S6). For reference, the polyclonal α-syn sdAb (poly-sdAb), from which monoclonals 2D8 and 2D10 were derived, had the same affinity rank as 2D10 with the highest affinity for the insoluble fraction from tg M83 mouse brain (*K*_D_ = 50.7 ± 6.9 nM), followed by the insoluble fraction from human LBD brain (*K*_D_ = 78.0 ± 22.1 nM), and lowest affinity for rec α-syn (*K*_D_ = 644.1 ± 200.1 nM) (Fig. 2F and fig. S6).

For comparison, their affinities for different α-syn species in the solution phase were determined (Fig. 2G and fig. S7). Here, 2D8 had comparable affinities for the different α-syn preparations (rec α-syn: *K*_D_ = 67.0 ± 18.5 nM, soluble fraction from LBD brain: *K*_D_ = 84.1 ± 12.4 nM, and soluble fraction from tg M83 mouse brain: *K*_D_ = 86.1 ± 2.6 nM). Likewise, 2D10’s affinities against the different α-syn species were comparable (rec α-syn: *K*_D_ = 51.7 ± 15.9 nM, soluble fraction from LBD brain: *K*_D_ = 46.7 ± 13.8 nM, and soluble fraction from tg M83 mouse brain: *K*_D_ = 29.0 ± 4.7 nM). The overall affinities of the poly-sdAb were less than the monoclonals but comparable between different α-syn preparations (rec α-syn: *K*_D_ = 144.1 ± 26.3 nM, soluble fraction from LBD brain: *K*_D_ = 103.8 ± 11.9 nM, and soluble fraction from tg M83 mouse brain: *K*_D_ = 126.0 ± 22.9 nM).

#### Anti-tau sdAbs

Selected representative clones were scaled up, purified, and screened for their ability to stain tau lesions on brain sections. One of them, 2B8, stood out for its ability to bind very strongly to tau tangles and pre-tangles (Fig. 2D), whereas staining of an adjacent brain section for phospho-tau with PHF1 revealed mostly mature tangles, dystrophic neurites, and possibly glial cells (Fig. 2E). To further test the specificity of the sdAb, we performed a dot blot assay using both human and mouse brain samples.

For the human samples, sdAb 2B8 bound strongly to two PHF samples and, to a moderate extent, to the LBD brain with more synucleinopathy (fig. S4E). Positive control anti-tau antibody PHF1 had a comparable reactivity as 2B8 to these three samples, which indicated that LBD brains had both tau and α-syn pathology (fig. S4F), which is common for LBD cases.

For the mouse samples, anti-tau sdAb 2B8 and positive control antibody PHF1 bound strongly to the tg P301L tau JNPL3 model brain supernatants and not to brain samples from WT controls or tg A53T α-syn M83 model mice (fig. S5, E and F). These results indicate the specificity of the 2B8 sdAb for tau pathology compared to synucleinopathy or other nonspecific targets in control tissue.

Subsequently, the affinity of the 2B8 sdAb for rec 2N4R tau and rec 1N4R hyperphosphorylated tau (p-tau) in their solution and solid phase was analyzed by BLI assays. Its affinity was high toward both of these tau species in solid (rec tau: *K*_D_ = 49.3 ± 17.0 nM and p-tau: *K*_D_ = 78.3 ± 10.7 nM; Fig. 2H and fig. S8, A and B) and solution phase (rec tau: *K*_D_ = 14.2 ± 0.4 nM and p-tau: *K*_D_ = 32.9 ± 17.0 nM; Fig. 2I, and fig. S8, C and D), indicating its suitability for subsequent in vivo imaging studies.

To clarify the binding sites of the sdAbs on target antigens, we also performed epitope mapping experiments via dot blot assays: 

#### Anti–α-syn sdAbs

**Fig. 3.**
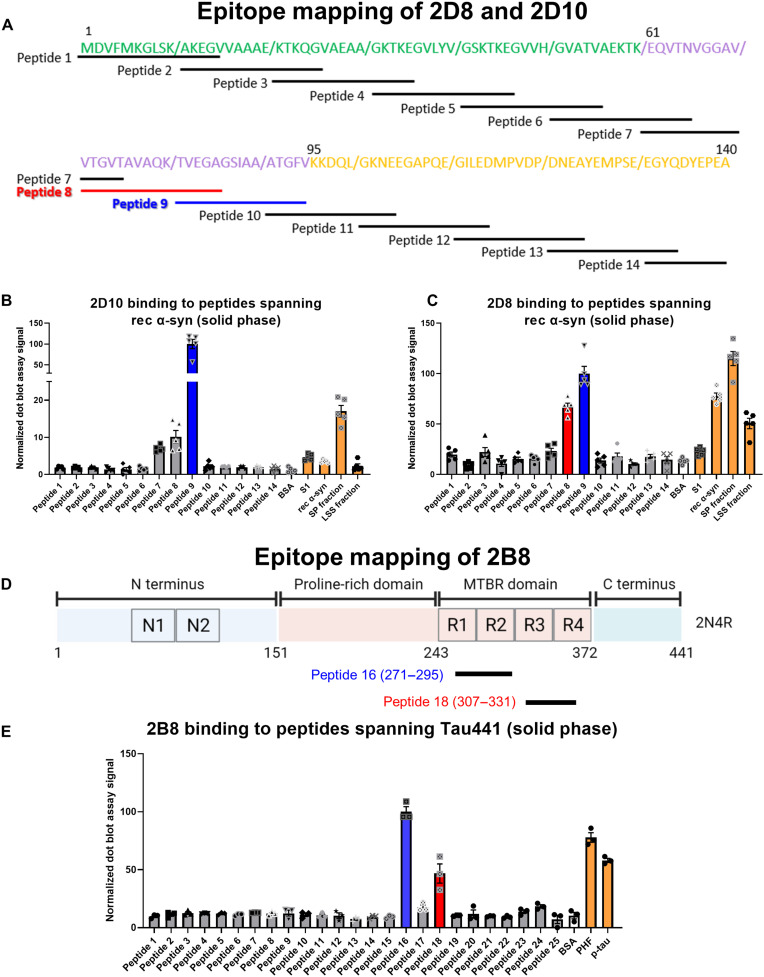
Epitope mapping of anti–α-syn versus anti-tau sdAbs. (**A**) Peptide library of α-syn. Amino acid sequences of 15-mer synthetic peptides (10-mer for peptide 14). Each peptide had a 5–amino acid overlap with the following peptide. Green depicts the N-terminal amphipathic repeat region (amino acids 1 to 60, membrane binding domain). Purple indicates the hydrophobic NAC domain (amino acids 61 to 94, promotes aggregation). Yellow shows the C-terminal acidic domain (amino acids 95 to 140). (**B** and **C**) Quantification of dot blot assay of 2D10 (B) and 2D8 (C). 2D10 bound mostly to peptide 9 (amino acids 81 to 95), whereas 2D8 reacted mainly with peptides 8 and 9 (amino acids 71 to 95). Positive control: α-syn preparations [soluble fraction (S1) from LBD brain, rec α-syn, insoluble SP fraction from tg-M83 mouse brain, and LSS soluble fraction from tg-M83 mouse brain]. Negative control: BSA. Each bar shows the mean normalized signal ± SD of five replicates. See fig. S9 for the dot blot assay images and table S3 for all of the peptide sequences. (**D**) The largest isoform of rec tau (2N4R, 441 amino acids) showing domain organization. (**E**) Quantification of dot blot assay on the binding of 2B8 to peptides covering all of the 441 amino acids of the tau protein. Each peptide within the library was 25 amino acids except the last one (peptide 25), which was 9 amino acids. Each peptide had a 7–amino acid overlap with the following peptide. Only peptides 16 (tau 271 to 295) and 18 (tau 307 to 331) displayed strong reactivity with 2B8. Positive control: PHF-enriched tau protein from human tauopathy brain and rec 1N4R isoform p-tau. Negative control: BSA. Each bar shows the mean normalized signal ± SD of three replicates. See fig. S10 (A and B) for the dot blot assay images and table S4 for all of the peptide sequences.

To identify the α-syn binding sites of the 2D8 and 2D10 sdAbs, their epitopes were mapped using 10 to 15 amino acid peptides covering overlapping regions of the whole 140–amino acid α-syn (table S3). We used bovine serum albumin (BSA) and α-syn preparations {human α-syn soluble fraction (S1) from LBD brain, rec α-syn, as well as insoluble [sarkosyl pellet (SP)] and soluble (LSS) fractions from a tg mouse M83 α-syn brain} as negative and positive controls, respectively, to validate the assay quality. Both sdAbs reacted well with some or most of the positive controls and not with the negative control. 2D10 reacted primarily with peptide 9 (α-syn 81 to 94), whereas 2D8 reacted most strongly with peptides 8 and 9 encompassing amino acids 71 to 94 of α-syn (Fig. 3, A to C, and fig. S9). These epitopes are within the hydrophobic NAC domain (amino acids 61 to 94) of α-syn, which is closely linked to α-syn aggregation (*19*).

#### Anti-tau sdAb

To determine the tau epitope that 2B8 binds to, we synthesized overlapping nonphosphorylated peptides covering the 441 amino acids of the tau protein with the internal peptides overlapping by seven amino acids (table S4). A dot blot assay revealed strong reactivity of 2B8 with peptides 16 (tau 271 to 295) and 18 (tau 307 to 331) and the positive controls PHF and p-tau (Fig. 3E and fig. S10, A and B). This binding was blocked or attenuated by preincubation of 2B8 with 10-fold molar excess of the peptides with the degree of peptide blocking fitting their degree of binding to 2B8 in the solid phase (fig. S10, C and D, and fig. S11A). A follow-up BLI assay showed high affinity of 2B8 for these tau peptides in solution (peptide 16: *K*_D_ = 12.1 ± 6.6 nM and peptide 18: *K*_d_ = 5.2 ± 1.7 nM; fig. S11, B and C). Notably, 2B8 has a higher affinity for peptide 18 than peptide 16 in the solution phase, whereas it binds better to peptide 16 in the solid-phase immunoblotting assay. Solid-phase BLI assay relies on biotinylating the bound target, so it is not suitable for small peptides such as these. The sequences of these two peptides are within the microtubule-binding repeat region of the tau protein and have substantial sequence homology (48%; table S4), which likely explains the binding to both peptides, although it could also indicate a discontinuous conformational epitope.

Overall, these affinity and epitope studies supported the suitability of the 2D8 and 2D10 sdAbs and, for comparison, their parent polyclonal sdAb for in vivo α-syn imaging and sdAb 2B8 for in vivo tau imaging. 

### Imaging studies by IVIS

#### Anti–α-syn sdAbs

##### Poly-sdAb

To determine whether the α-syn sdAbs could detect α-syn deposits in vivo in intact animals, M83 α-syn A53T synucleinopathy mice (7 months of age) received a single intravenous injection of the poly-sdAb derived from the llama immunized with rec α-syn. In an initial proof-of-concept experiment, it was labeled with a near-infrared (NIR) tag, VivoTag 680XL, to allow its in vivo detection by the In Vivo Imaging System (IVIS) through the intact head (the fur was shaved, but no skin incision was made; fig. S12A). A strong brain signal, which peaked within the first hour, was detected in the mice (fig. S12, B and C), with some of the signal remaining up to 96 hours later (fig. S13A). The brain signal coincided with a strong signal from the spinal cord in the M83 mice, which in this model has extensive α-syn pathology (*20*). An increasing signal from the kidneys was evident in the animals during the first imaging session. This is as expected because the size of the sdAbs is below the cutoff for kidney reabsorption. After imaging, their brains were extracted to determine how well the IVIS brain signal correlated with soluble and insoluble α-syn in the brain. A very strong correlation was observed between the cumulative [area under the curve (AUC)] IVIS signal within 2 hours and α-syn, including both insoluble α-syn (*r* = 0.904, *P* = 0.0133) and soluble α-syn (*r* = 0.836, *P* = 0.0380) (fig. S12, D to G).

##### 2D10 and 2D8

**Fig. 4.**
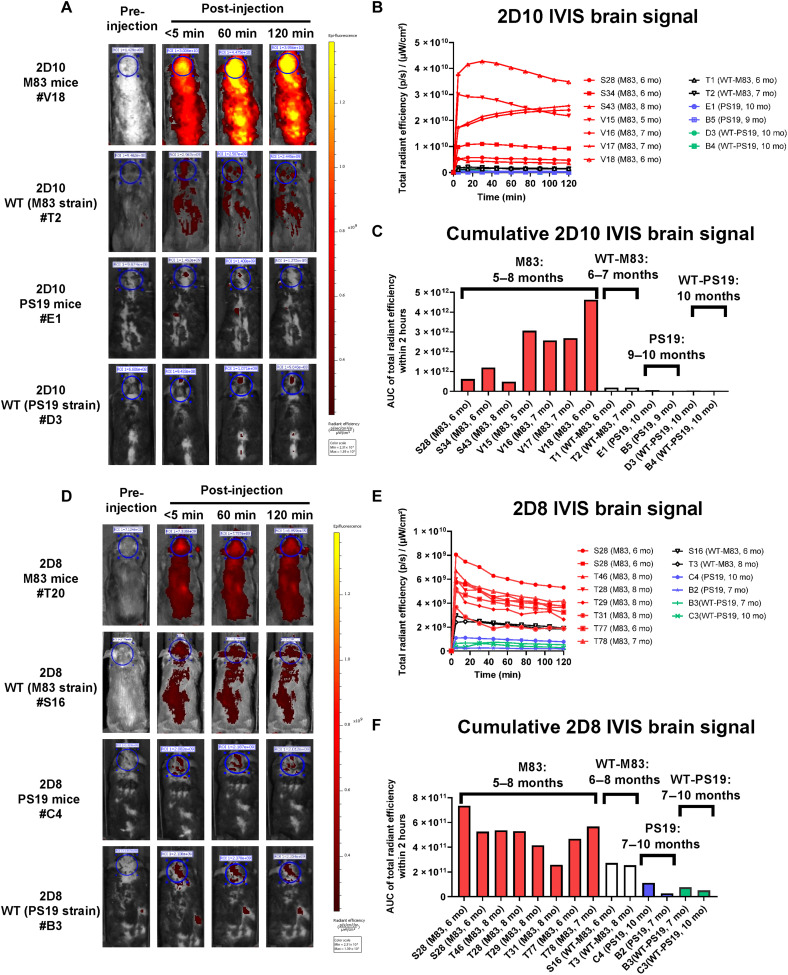
In vivo imaging in M83 synucleinopathy and control mice. (**A**) Representative images of brain signal following intravenous injection with labeled sdAb-2D10 (10 mg/kg). The images were recorded before injection and after injection at <5, 60, and 120 min. The first row shows a homozygous tg-M83 synucleinopathy mouse. The second, third, and fourth rows depict a WT mouse (M83 strain background), heterozygous PS19 (tau P301S) tauopathy tg-mouse, and its WT control (PS19 strain background) littermate under the same experimental conditions as the synucleinopathy mice. Strong brain (yellow in ROI, circle) and peripheral (spinal cord) signal was detected in the M83 mice, whereas limited signal was detected in the controls after 2D10 injection. The scale bar shows the maximum pixel intensity. (**B** and **C**) Quantitative analysis of brain signal over time (B) and cumulatively within 2 hours after injection (C). The AUC is the total radiant efficiency of summed pixel intensity. (**D**) Representative images of brain signal following intravenous injection with labeled sdAb-2D8 (10 mg/kg). The images were recorded before injection and after injection at <5, 60, and 120 min. The first row shows a homozygous tg-M83 synucleinopathy mouse. The second, third, and fourth rows depict a WT mouse (M83 strain background), heterozygous PS19 (tau P301S) tauopathy tg mouse, and its WT control (PS19 strain background) littermate under the same experimental conditions as the synucleinopathy mice. Moderate brain signal was detected in the M83 mice, whereas limited signal was detected in the controls after 2D8 injection. The scale bar shows the maximum pixel intensity. (**E** and **F**) Quantitative analysis of IVIS brain signal over time (E) and cumulatively within 2 hours after injection (F). The AUC is the total radiant efficiency of summed pixel intensity.

On the basis of this promising finding and after characterization of several sdAbs, 2D8 and 2D10 were selected for additional IVIS studies based on their high affinity (low nanomolar) for solid-phase insoluble α-syn, as detailed above (Fig. 2, F and G). To determine whether the α-syn 2D10 and 2D8 sdAb could detect α-syn deposits in vivo in intact animals, homozygous synucleinopathy M83 mice (6 to 8 months of age) received a single intravenous injection of either 2D10 or 2D8 labeled with the same NIR tag, VivoTag 680XL, as the poly-sdAb, to allow their in vivo detection by IVIS through the intact head (Fig. 4). As for poly-sdAb, the brain signal coincided with a signal from the spinal cord in the M83 mice.

For 2D10, a strong signal was detected in the M83 α-syn mice but only a very weak signal in WT or tauopathy mice (Fig. 4, A to C). The first row of Fig. 4A shows a homozygous tg-M83 synucleinopathy mouse with α-syn brain signal increasing up to 60 min after injection [yellow in the region of interest (ROI), circle)]. The signal was detected throughout the body with the highest intensity in the brain. Limited brain or peripheral signal was detected in the controls, including WT mouse (M83 strain background), heterozygous PS19 (tau P301S) tauopathy tg-mouse, and its WT control (PS19 strain background) after 2D10 injection (second, third, and fourth rows of Fig. 4A). Brains from mice *S28* and *V15-18* were removed at 2 hours and processed for confocal analyses of the sdAb-680 signal and its colocalization with α-syn and markers of the endosomal-lysosomal system as described below (see the “Histological analysis of sdAb brain signal” section). In the remaining synucleinopathy mice, 41 to 65% of the sdAb signal remained 24 hours later and was mostly cleared within a few days (see fig. S13B). As shown in the representative images in Fig. 4A, a much stronger brain 2D10 signal was seen in the homozygous M83 mice, with limited signal in the control groups, including both PS19 tauopathy mice and WT mice (both M83 and PS19 strain background), supporting the specificity of the 2D10 imaging probe for α-syn lesions.

The tagged 2D8 sdAb displayed a similar pattern, showing a robust signal in the M83 α-syn mice and a relatively weak signal in the WT and tauopathy mice (Fig. 4, D to F). The first row of Fig. 4D shows a homozygous tg-M83 synucleinopathy mouse with α-syn brain signal increasing quickly and remaining stable throughout the 120 min. The signal was detected throughout the body with the highest intensity in the brain (ROI, circle). In addition, limited brain or peripheral signal was detected in the controls, including WT mouse (M83 strain background), heterozygous PS19 (tau P301S) tauopathy tg-mouse, and its WT control (PS19 strain background) after 2D8 injection (second, third, and fourth rows of Fig. 4D). Brains from mice *T20-21* were removed at 2 hours and processed for confocal analyses of the sdAb-680 signal and its colocalization with α-syn and markers of the endosomal-lysosomal system, as described below (see the “Histological analysis of sdAb brain signal” section). In the remaining synucleinopathy mice, 32 to 61% of the sdAb signal remained 24 hours later and was mostly cleared within a few days (see fig. S13C). As shown in the representative images in Fig. 4D, a stronger brain 2D8 signal was seen in the homozygous M83 mice, with limited signal in the PS19 tauopathy mice and WT (both M83 and PS19 strain background), supporting the selectivity of the 2D8 imaging probe for α-syn lesions.

Overall though, 2D8 did give a weaker signal than 2D10 and did not appear to be as specific. Notably, no sdAb signal was detected in brain sections of these WT mice injected with either 2D10 or 2D8 (figs. S15 and S16). See table S5 for a list of the imaged mice and their ages.

**Fig. 5.**
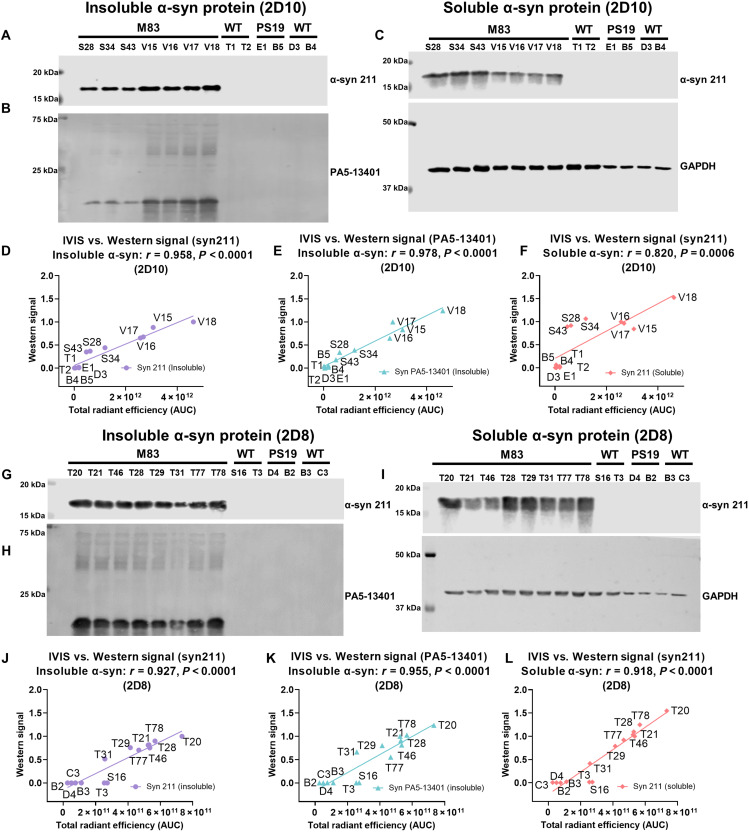
Correlation of insoluble and soluble α-syn brain levels with IVIS brain imaging signal for 2D10 and 2D8. *sdAb 2D10*: Western blot depicting SP insoluble fraction reacted with (**A** and **D**) antibody syn 211: *r* = 0.958, *P* < 0.00001 and (**B** and **E**) antibody PA5-13401: *r* = 0.978, *P* < 0.00001, and soluble fraction reacted with (**C** and **F**) antibody syn 211: *r* = 0.820, *P* = 0.0006, in M83 mice (*S28, S34, S43,* and *V15-18*), WT mice (M83 strain) (*T1-2*), PS19 tauopathy mice (*E1* and *B5*), and WT mice (PS19 strain) (*D3* and *B4*). For correlation analysis (F), soluble α-syn levels were normalized to glyceraldehyde-3-phosphate dehydrogenase (GAPDH) levels. *sdAb 2D8*: Western blot depicting SP insoluble fraction reacted with (**G** and **J**) antibody syn 211: *r* = 0.927, *P* < 0.0001 and (**H** and **K**) antibody PA5-13401: *r* = 0.955, *P* < 0.00001, and soluble fraction reacted with (**I** and **L**) antibody syn 211: *r* = 0.918, *P* < 0.0001, in M83 mice (*T20, T21, T46, T28, T29, T31, T77,* and *T78*), WT (M83 strain) mice (*S16* and *T3*), and PS19 tauopathy mice (*D4 and B2*), and WT mice (PS19 strain) (*B3* and *C3*). For correlation analysis (L), soluble α-syn levels were normalized to GAPDH levels.

After imaging, brains were extracted to determine how well the IVIS brain signal correlated with soluble and insoluble α-syn in the brain (Fig. 5). For 2D10, the cumulative IVIS signal (AUC) correlated strongly with both insoluble (*r* = 0.958, *P* < 0.0001; Fig. 5, A and D) and soluble α-syn (*r* = 0.820, *P* = 0.0006; Fig. 5, C and F) using monoclonal antibody syn 211. For 2D8, the correlation between the IVIS signal and both insoluble (*r* = 0.927, *P* < 0.0001; Fig. 5, G and J) and soluble α-syn (*r* = 0.918, *P* < 0.0001; Fig. 5, I and L) was equally strong. These analyses are based on quantifying the α-syn monomer band since high–molecular weight bands showed limited, if any, reactivity with the syn 211 antibody. For further confirmation, blots with the insoluble fraction were reacted with the polyclonal α-syn antibody, PA5-13401, which detects both α-syn monomers and higher–molecular weight bands of α-syn. The sum of all of these bands was calculated and correlated strongly with the IVIS brain signal of both 2D10 (*r* = 0.978, *P* < 0.0001; Fig. 5, B and E) and 2D8 (*r* = 0.955, *P* < 0.0001; Fig. 5, H and K). For complete α-syn Western blots and bands quantified, see fig. S21.

#### Anti-tau sdAb

**Fig. 6.**
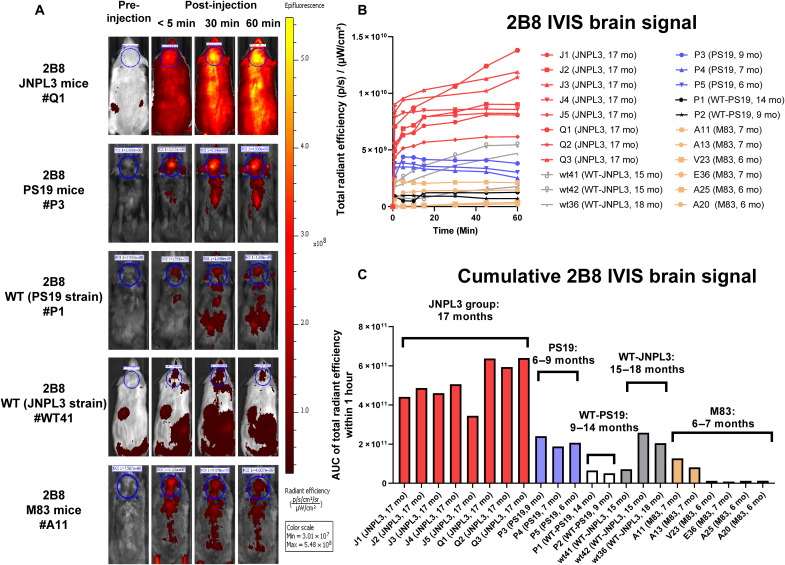
In vivo imaging in JNPL3 and PS19 tauopathy mice and control mice. (**A**) Representative images of brain signal following intravenous injection with labeled sdAb-2B8 (10 mg/kg). The images were recorded before injection and after injection at <5, 30, and 60 min. The first row shows images of a homozygous JNPL3 tauopathy tg mouse (17 months) with brain signal increasing rapidly during the 60 min (yellow in ROI, circle). The second row contains images of a heterozygous PS19 tauopathy tg mouse (9 months). The third and fourth rows depict WT mice of PS19 (14 months) and JNPL3 (18 months) strain backgrounds, and the fifth row shows a homozygous M83 synucleinopathy tg mouse (7 months) under the same experimental conditions as the tauopathy mice. The strongest signal was detected in the older homozygous JNPL3 mice, whereas the younger heterozygous PS19 mice emitted a weaker signal. Limited brain or peripheral signal was detected in the control mice after 2B8 injection. The scale bar shows maximum pixel intensity. (**B** and **C**) Quantitative analysis of IVIS brain signal over time (B) and cumulatively (C). The AUC is total radiant efficiency of summed pixel intensity. Note that no sdAb signal was detected in brain sections of the old WT mice of the JNPL3 strain background that had a gradual increase in their brain signal, which notably remained below all of the JNPL3 mice (see fig. S17).

To determine whether the tau 2B8 sdAb could detect tau deposits in vivo in intact animals, homozygous JNPL3 tauopathy mice (17 months of age, *n* = 8) and heterozygous PS19 mice (6 to 9 months, *n* = 3) received a single intravenous injection of 2B8 labeled with an NIR tag, VivoTag 680XL, to allow its detection by the IVIS through the intact head (Fig. 6, A to C). A strong signal, which typically peaked within the first hour, was detected in the mice, with some of it remaining up to at least 100 hours later (fig. S14). The first row of Fig. 6A shows images of a homozygous JNPL3 tauopathy tg mouse (17 months) with tau brain signal increasing rapidly during the 60 min (yellow in ROI, circle). The signal was detected throughout the body with the highest intensity in the brain. The second row contains images of a heterozygous PS19 tauopathy tg mouse (9 months). Again, the most intense signal was detected in the brain, but these younger mice of a different tauopathy model had fewer signals than JNPL3 mice in both brain and periphery. The third and fourth rows depict WT mice of PS19 (14 months) and JNPL3 (18 months) strain backgrounds, and the fifth row shows a homozygous M83 synucleinopathy tg mouse (7 months) under the same experimental conditions as the tauopathy mice. Limited brain or peripheral signal was detected after the 2B8 injection. The scale bar shows the maximum pixel intensity.

**Fig. 7.**
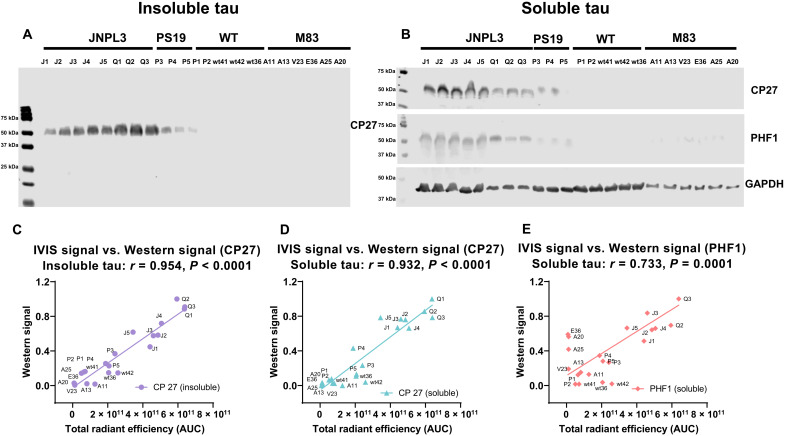
Correlation of 2B8 sdAb IVIS brain signal with brain levels of insoluble and soluble tau. (**A**) A Western blot depicting insoluble human tau protein (SP reacted with CP27) in tauopathy JNPL3 and PS19 mice (*J1* to *J5*, *Q1* to *Q3*, and *P3* to *P5*) and lack thereof in WT (*P1*, *P2*, *wt41*, *wt42*, and *wt36*) and synucleinopathy M83 mice (*A11*, *A13*, *V23*, *E36*, *A25*, and *A20*). (**B**) Western blots depicting soluble human tau (CP27) and phospho-tau (PHF1) in the same animals as in (A). For correlation analysis (D and E), these levels were normalized to GAPDH levels. (**C** to **E**) Correlation between overall IVIS brain signal with insoluble tau [(C): CP27: *r* = 0.954, *P* < 0.0001] and soluble tau within the brain [(D): CP27: *r* = 0.932, *P* < 0.0001; (E): PHF1: *r* = 0.733, *P* = 0.0001].

The brain signal coincided with a strong signal from the spinal cord in the JNPL3 mice, which in this model has extensive tau pathology. Notably, the signal was highly specific to the tauopathy mice, with the strongest signal detected in the older homozygous JNPL3 mice, whereas the younger heterozygous PS19 mice emitted a weaker signal. Only a minimal signal was detected in WT mice of the two strain backgrounds (*n* = 5) and in the M83 synucleinopathy model (*n* = 6) (Fig. 6, A to C). Brains from mice *Q1* to *Q3* were removed at 1 hour and processed for confocal analyses of the sdAb-680 signal and its colocalization with various tau epitopes and markers of the endosomal-lysosomal system, as described below (see the “Histological analysis of sdAb brain signal” section). In the remaining tauopathy mice, 50 to 68% of the sdAb signal remained 24 hours later and was mostly cleared within a few days (see fig. S14). See table S6 for a list of the imaged mice and their ages. Two of the old WT mice of the JNPL3 strain background (15 and 18 months) had a gradual increase in their brain signal, but it was always less than in all of the age-matched JNPL3 tauopathy (17 months). This may relate to age-related leakage of the blood-brain barrier. Notably, no sdAb signal was detected in the brain sections of these WT mice (see fig. S19). The PS19 tauopathy mice (6 to 9 months) also had a much higher sdAb signal compared to WT animals of the same strain background, including a 14-month-old WT mouse. Of the six M83 α-syn mice (6 to 7 months), only two had a weak signal above background levels (Fig. 6). Following imaging, all animals were perfused and the brains were extracted to determine how well the IVIS brain signal correlated with soluble and insoluble tau in the brain (Fig. 7). Western blotting was performed using CP27, an antibody recognizing human tau, and PHF1 recognizing tau phosphorylated at Ser396 and Ser404. The cumulative IVIS signal (AUC) correlated strongly with both insoluble (CP27: *r* = 0.954, *P* < 0.0001; Fig. 7, A and C) and soluble human tau (CP27: *r* = 0.932, *P* < 0.0001; PHF1: *r* = 0.733, *P* = 0.0001; Fig. 7, B, D, and E). For complete tau Western blots and the bands quantified, see fig. S22.

### Histological analysis of sdAb brain signal

We then performed additional experiments to confirm that the anti–α-syn sdAbs (2D8 and 2D10) or the anti-tau sdAb (2B8) were taken up into the brain, internalized by neurons, and bound to pathological α-syn or tau, respectively.

#### Anti–α-syn sdAb

**Fig. 8.**
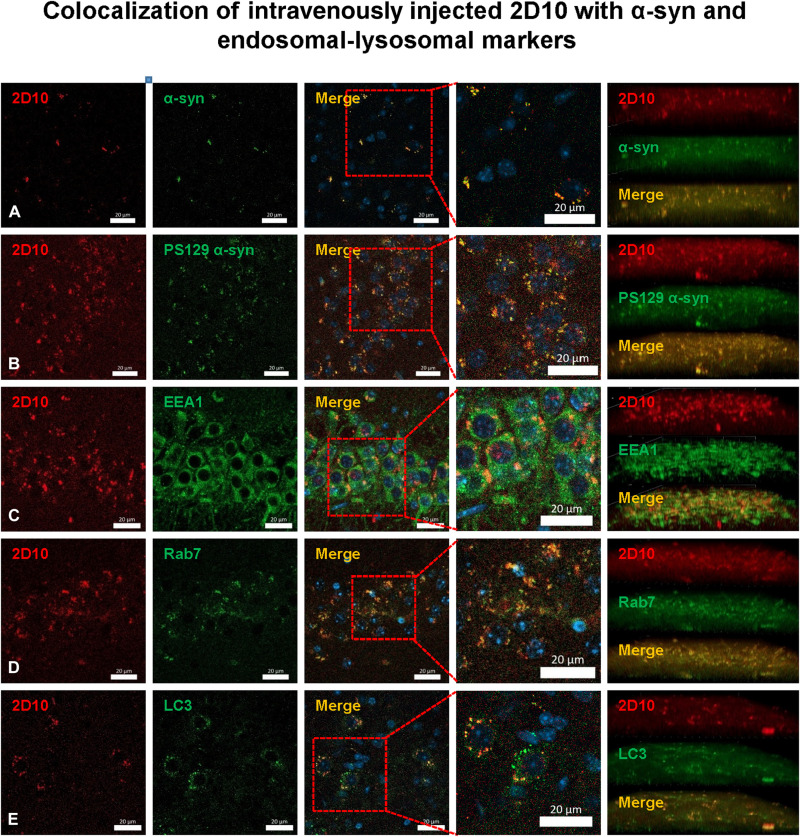
Colocalization of intravenously injected 2D10 sdAb with α-syn and markers of endosomes, lysosomes, and autophagosomes in M83 α-syn mice. NIR dye–labeled sdAb 2D10 was injected intravenously; the brains were perfused with PBS and removed 2 hours after injection, postfixed, sectioned, and stained with a nuclear stain [4′,6-diamidino-2-phenylindole (DAPI), blue] and an antibody against (**A**) total α-syn (α-syn 211) or (**B**) phosphorylated α-syn (phospho-serine 129). Merged images from representative M83 α-syn mouse *V18* revealed that sdAb 2D10 entered the brain following intravenous injection and was taken up into neurons, where it bound to pathological α-syn protein in the endosomal-lysosomal system as per sections stained with an antibody against (**C**) early endosomes (EEA1) or (**D**) late endosomes/lysosomes (Rab7), and (**E**) autophagosomes (LC3). Z stacks (right) confirm extensive colocalization as quantified by Manders’ coefficients: 2D10/syn 211, 0.770; 2D10/syn p-serine 129, 0.790; 2D10/EEA1, 0.963; 2D10/Rab7, 0.746; 2D10/LC3, 0.892. Scale bars, 20 μm. For representative images of lack of 2D10 signal in WT mice and tauopathy mice, see figs. S15 and S17.

**Fig. 9.**
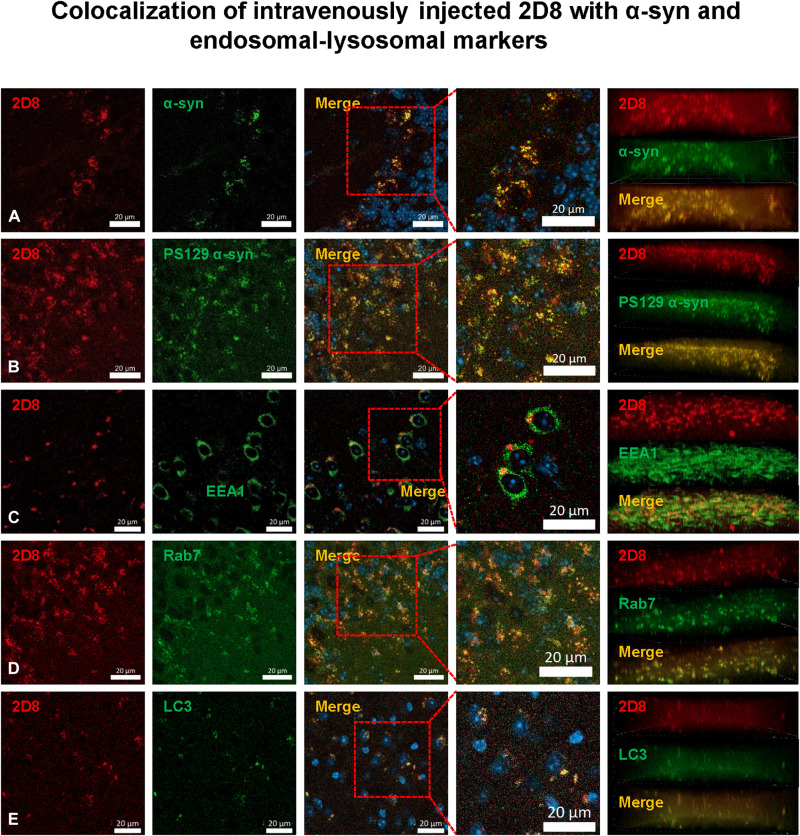
Colocalization of intravenously injected 2D8 sdAb with α-syn and markers of endosomes, lysosomes, and autophagosomes in M83 α-syn mice. NIR dye–labeled sdAb 2D8 was injected intravenously; the brains were perfused with PBS and removed 2 hours after injection, postfixed, sectioned, and stained with a nuclear stain (DAPI), and an antibody against (**A**) total α-syn (α-syn 211) or (**B**) phosphorylated α-syn (phospho-serine 129). Merged images from representative M83 α-syn mouse *T20* revealed that sdAb 2D8 entered the brain following intravenous injection and was taken up into neurons, where it bound to pathological α-syn protein in the endosomal-lysosomal system as per sections stained with an antibody against (**C**) early endosomes (EEA1 or (**D**) late endosomes/lysosomes (Rab7), and (**E**) autophagosomes (LC3). Z stacks (right) confirm extensive colocalization as quantified by Manders’ coefficients: 2D8/syn 211, 0.640; 2D8/syn p-serine 129, 0.802; 2D8/EEA1, 0.786; 2D8/Rab7, 0.815; 2D8/LC3, 0.721. Scale bars, 20 μm. For representative images of lack of 2D8 signal in WT mice and tauopathy mice, see figs. S16 and S18.

To verify that the sdAbs were taken up into the brain, into neurons, and that they are bound to pathological α-syn, selected brains were extracted 2 hours following the intravenous injection (V15-18 and T20), and their right hemisphere was immersion fixed in paraformaldehyde, processed, sectioned, and stained with α-syn antibodies and markers of endosomes, autophagosomes, and lysosomes. Both of the monoclonal sdAbs were readily detected in the brain, inside neurons bound to α-syn (total and p-S129) within the early endosomes (EEA1), late endosomes/lysosomes (Rab7), and autophagosomes (LC3) (Figs. 8 and 9). In contrast, no sdAb signal was detected in brain sections from the sdAb-injected WT mice that were treated in the same manner (figs. S15 and S16) or tauopathy mice (figs. S17 and S18). These findings confirm the suitability of these two sdAbs as potential imaging probes to detect synucleinopathies in intact live subjects. Specifically, 2D10 sdAb was readily detected in the brain, inside neurons bound to α-syn (Manders’ coefficients = 0.770 to 0.790) within the early endosomes, late endosomes/lysosomes, and autophagosomes (Manders’ coefficients = 0.963, 0.746, and 0.892, respectively). Likewise, the 2D8 sdAb was readily detected in the brain, inside neurons bound to α-syn (Manders’ coefficients = 0.640 to 0.802) within the early endosomes, late endosomes/lysosomes, and autophagosomes (Manders’ coefficients = 0.786, 0.815, and 0.721, respectively). Together, these findings indicate that 2D10 and 2D8 are highly suitable as diagnostic imaging agents to detect α-syn aggregates in intact living subjects.

#### Anti-tau sdAb

**Fig. 10.**
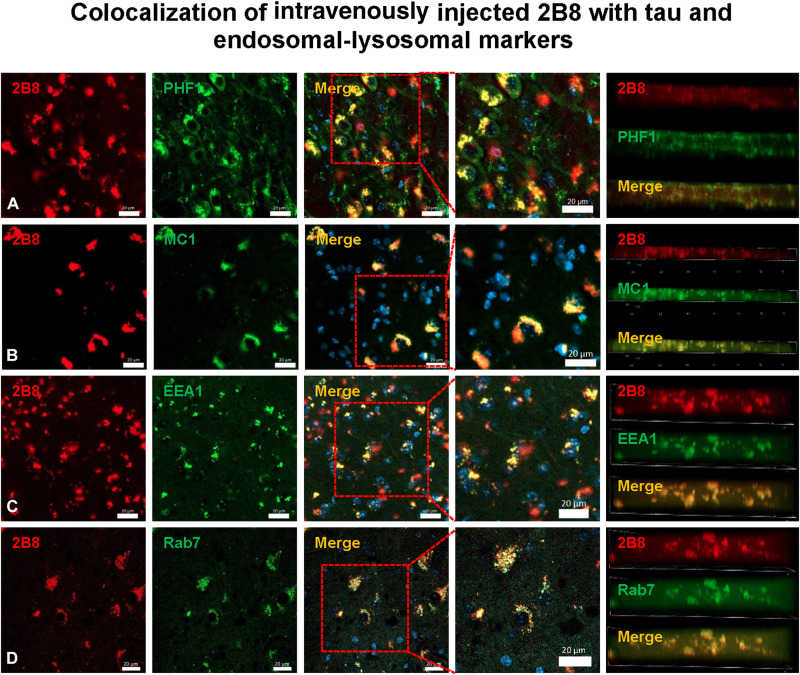
Brain sections from 2B8 injected tauopathy mice stained with tau and endosomal-lysosomal markers to assess the presence of 2B8 within the brain and its cellular location. NIR dye–labeled sdAb 2B8 was injected intravenously; the brains were perfused with PBS and removed 1 hour after injection, postfixed, sectioned, and stained with a nuclear stain (Hoechst), and an antibody against (**A**) p-tau (PHF1) or (**B**) a conformational tau epitope (MC1). The representative images are from JNPL3 mouse *Q1*. Merged coronal images revealed that sdAb 2B8 enters the brain following intravenous injection and is taken up into neurons, where it binds to pathological tau protein in the endosomal-lysosomal system as per sections stained with an antibody against (**C**) early endosomes (EEA1) or (**D**) late endosomes/lysosomes (Rab7). Z stacks (right) confirm extensive colocalization of 2B8 with the different markers as quantified by Manders’ coefficients in the third panel from the left (2B8/PHF1, 0.847; 2B8/MC1, 0.916; 2B8/EEA1, 0.863; 2B8/Rab7, 0.708). Scale bars, 20 μm. For representative images of lack of 2B8 signal in WT mice and synucleinopathy mice, see figs. S19 and S20.

Selected brains were extracted 1 hour following the intravenous injection (Q1 to Q3) and their right hemisphere was immersion fixed in paraformaldehyde, processed, sectioned, and stained with tau antibodies against different epitopes [phospho-tau (PHF1) and misfolded tau (MC1)], as well as markers of early (EEA1) and late endosomes and lysosomes (Rab7; Fig. 10). 2B8 sdAb was readily detected in the brain, colocalized with neuronal tau (Manders’ coefficients = 0.847 and 0.916), and was primarily found within the early endosomes and late endosomes/lysosomes (Manders’ coefficients = 0.863 and 0.708). In contrast, limited, if any, 2B8 signal was detected in WT animals (fig. S19) or synucleinopathy mice (fig. S20). Overall, these findings indicate that 2B8 sdAb is highly suitable as an imaging probe to detect tau lesions in intact living subjects.

## DISCUSSION

In this study, we have developed sdAb-based imaging probes that, following their intravenous injection, bind with high sensitivity and specificity to α-syn versus tau lesions, respectively, and allow their specific in vivo detection in intact animals. The two anti–α-syn sdAbs consistently showed strong brain signals via the intact head after peripheral injection in M83 α-syn mice but not in tauopathy or WT mice. Their brain signals correlated strongly with insoluble α-syn brain levels analyzed postmortem (syn 211: *r* = 0.958 and 0.927; PA5-13401: *r* = 0.978 and 0.955 respectively, *P* < 0.0001). Signal correlation with soluble α-syn levels was less for 2D10 but comparable for 2D8 and highly significant for both sdAbs (2D10: *r* = 0.820, *P* = 0.0006; 2D8: *r* = 0.919, *P* < 0.0001). These two sdAbs were identified via solid-phase panning, which likely relates to their comparable recognition of insoluble α-syn. Likewise, the anti-tau sdAb, 2B8, gave a strong brain signal in two tauopathy tangle mouse models but not in WT or M83 α-syn mice. Its in vivo brain signal correlated strongly with insoluble (CP27: *r* = 0.954, *P* < 0.0001) and soluble tau levels (CP27: *r* = 0.932, *P* < 0.0001 and PHF1: *r* = 0.733, *P* = 0.0001) in the brains of the animals.

Confocal postmortem analyses of the animals’ brains revealed that the sdAbs were mainly found within neurons, and their colocalization with various markers was quantified by Manders’ coefficients. The anti–α-syn sdAbs colocalized extensively and specifically with α-syn [2D10: 0.770 (α-syn) and 0.790 (α-syn p-S129); 2D8: 0.640 (α-syn) and 0.802 (α-syn p-S129)]. Likewise, the anti-tau sdAb, 2B8, colocalized with intraneuronal tau [0.847 (PHF1) and 0.916 (MC1)]. The interaction took place primarily within the endosomal-lysosomal system [2D10: 0.963 (EEA1) and 0.746 (Rab7); 2D8: 0.786 (EEA1) and 0.815 (Rab7); 2B8: 0.863 (EEA1) and 0.708 (Rab7)] and within autophagosomes (LC3) as examined for the anti–α-syn sdAbs (2D10: 0.892; 2D8: 0.721). Notably, no sdAb signal was detected in brain sections from control animals without intraneuronal deposits of α-syn or tau, respectively.

We and others have observed similar neuronal uptake, and when examined, the intracellular distribution of certain antibodies and their Fab or scFv fragments after peripheral administration in models of tauopathy, synucleinopathy, or other diseases with intracellular protein aggregates or after dosing into the media in brain slice or cellular culture models (*18*, *21*–*31*). Antibody-based imaging probes are much more specific than β sheet dyes, but whole antibodies are unlikely to get into the brain and into cells in sufficient quantities to reliably allow imaging these intracellular lesions in intact living subjects. On the other hand, sdAbs have high affinity for their target while being only ^1^/_10_ the size of whole antibodies (about 15 versus 150 kDa) (*32*). This results in greater brain uptake and distribution and, therefore, enhanced sensitivity to detect their target. They also have a more suitable shorter half-life as imaging probes because their small size is below the cutoff for kidney reabsorption.

Currently, no specific α-syn imaging probes are approved for clinical use. Development of small molecules with such properties has been limited because of cross-reactivity with other β sheet peptides/proteins (*15*–*17*). We are not aware of other reports showing the diagnostic promise of sdAbs targeting α-syn. In addition to our low–nanomolar affinity anti–α-syn sdAb clones, a couple of sdAbs targeting α-syn (*K*_D_ = 10 to 547 nM against different α-syn preparations) have been generated and characterized by others in various assays but not yet as diagnostic imaging agents (*33*, *34*).

The hope for tau-based ligands is that they will allow better insight into the status and progression of neurodegeneration than Aβ ligands because tau lesions correlate much better with cognitive impairment than Aβ deposits (*35*). The current ligands that are being evaluated clinically are all β sheet binders and, therefore, have some affinity for various amyloids (*13*, *14*). Their first generation only detected tau lesions in AD and not in primary tauopathies, and many turned out to have high affinities for nonamyloids, such as monoamine oxidase B (*13*, *14*, *36*). Their second generation is improved, and at least some of those recognize tau aggregates in primary tauopathies (*13*, *14*). However, because all were selected on the basis of their β sheet binding, they will never reach the specificity of antibodies. These small molecules are all being examined for PET, which gives a reasonable three-dimensional (3D) view of their binding within the brain. Such detailed viewing is necessary because of their nonspecific binding in various brain regions. In addition, in contrast to β sheet–bound dyes, probes derived from antibodies against different epitopes of the target protein can provide a detailed pathological profile of those proteins in each individual. This information can then guide therapeutic options, which may include immunotherapies against the same epitopes detected by imaging.

It is quite clear that whole antibodies do not cross the blood-brain barrier in sufficient quantities to be useful for PET scans, but some success has been obtained in PET imaging of Aβ deposits by attaching an anti-Aβ F(ab)_2_ to a transferrin antibody to facilitate its entry into the brain (*37*). However, that approach does not work for intraneuronal α-syn and then probably not for intraneuronal tau (*38*). Since sdAbs are about ^1^/_10_ and ^1^/_7_ the size of whole antibodies or F(ab)_2_, respectively, and therefore get into the brain in much larger quantities, such engineering may not be necessary for sdAbs. Furthermore, the outstanding correlation between the IVIS brain signal of the sdAb and α-syn versus tau levels in the brain makes it likely that 2D detection with this probe may be sufficient for the diagnosis of their pathological burden in humans. With regard to potential safety concerns, because of their small size, sdAbs are also much less immunogenic than whole antibodies. If further modification is needed to avoid immunogenicity in longitudinal studies, the sdAbs can also easily be humanized.

One sdAb derived from llama, caplacizumab, is clinically approved to treat a blood disorder (*39*), and sdAbs against various misfolded protein targets have been assessed in experimental models, particularly in a gene therapy–related context (*40*). However, only a few anti-tau or anti–α-syn sdAbs have been described in the literature. In addition to the two α-syn sdAbs mentioned above (*33*, *34*), a few more have recently been reported but none have been examined as in vivo diagnostic imaging probes following an intravenous injection (see note added in proof). One anti-tau sdAb was generated from pooled phage display libraries from a couple of immunized llamas that received tangle-enriched AD extracts or a phospho-tau-Ser422 peptide (*41*). It bound to tau lesions in the brains of two rTg4510 tauopathy mice following an intravenous injection as visualized by two-photon imaging following a craniotomy. Another one tau-targeting sdAb was derived from a synthetic phage display library and showed some therapeutic efficacy following its intracerebral injection in a lentivirus (*42*). The extent of its brain entry following intravenous injection or its feasibility as a diagnostic imaging probe have not been reported. In addition, we recently reported on the efficacies of two anti-tau sdAbs, including 2B8, in clearing pathological tau and preventing its toxicity in culture and in tg tauopathy mice (*43*).

There are three types of sdAb libraries. Immune libraries are obtained by immunizing an animal like the llama that generates such antibodies, and then the library is generated as described in our approach. Its main advantage is sdAbs with high binding affinity for the immunogen. Naïve libraries are generated by following the same procedure in an unimmunized animal. This approach requires a large pool of blood to capture the high diversity of autoantibodies that are generally of low affinity for various targets. Synthetic libraries are the third type. Unlike sdAbs from animal hosts, each of which has its own framework region, all sdAbs from a synthetic nanobody library need to share the same framework region sequence. It needs to be stable, well-expressed, and highly versatile. Once this sequence has been selected, the CDR regions have to be designed, which typically involves randomization, and this type of library is in many ways comparable to the naïve library since it is not designed for one antigen or epitope but rather to have a diverse binding to various antigens. It, therefore, needs to be large, which requires considerable effort. In addition, undesirable frameshift mutations are common in synthetic libraries, which diminishes their quality (*44*).

IVIS imaging is more cost-effective than PET studies and involves simpler probe preparation without complex radiolabeling procedures. Thus, IVIS imaging is ideal for probe development and selecting ligands for subsequent PET studies. Our study also suggests that it may be an effective method for monitoring the development of α-syn versus tau pathology and for screening treatment-mediated clearance of these proteins in longitudinal studies, with each animal serving as its own control. It is well established that pathological α-syn or tau burden can vary substantially between mice of the same model, even at the same age. Therefore, therapy studies in these models typically require a rather large cohort to be able to detect differences between treatment and control groups. As per the strong correlation of brain signal with α-syn and tau levels on Western blots, IVIS imaging will allow assigning animals into groups with a similar range of α-syn and tau burden, which will increase the statistical power of the study and therefore require fewer animals. IVIS imaging may also be suitable to monitor treatment-mediated clearance of α-syn or tau aggregates in longitudinal studies, with each animal serving as its own control. As mentioned above, this or a similar type of 2D imaging may actually be sufficient to assess pathological protein burden in humans because of the superior specificity of antibody-derived probes for their target over small molecules that selectively bind to more generic β sheet structures.

Although this particular NIR imaging approach has not been performed yet in humans, there is reason to be optimistic regarding its translatability. A different type of noninvasive functional NIR imaging has been conducted in humans for many years, which has primarily been focused on NIR absorption of oxyhemoglobin and deoxyhemoglobin (*45*, *46*). This allows the assessment of tissue oxygenation within cortical brain regions, which can then be interpreted as brain activity. These human NIR imaging systems rely on several emitters and detectors of NIR light that are placed on the head of the person. In our animal studies, the emitter is the probe that we injected into the mouse, and the detector is the IVIS camera. Given that the technology to detect NIR signals is already well established, it should be relatively straightforward to translate this to humans. It may simply require building larger IVIS systems for human use. Notably, most of the NIR imaging in use today is within the 700- to 1000-nm range (NIR-I), but there is an active development in generating emitters or tags in the 1000- to 1700-nm range (NIR-II), which will allow deeper reach into the tissue, such as deep cortical regions or even some subcortical brain areas, and thereby provide better diagnostic insight (*47*, *48*). With this in mind, the current NIR-I chemical tag bound to the sdAbs may be sufficient to detect cortical synucleinopathy or tauopathy as found in LBD versus various tauopathies, respectively, or peripheral synucleinopathy as found in multiple system atrophy. LDB can occur with AD pathology, and these probes may then allow in vivo evaluation of the degree of LBD pathology in those individuals, in conjunction with imaging for tau and Aβ lesions. The second-generation NIR-II tags would then further improve this diagnostic approach.

Future studies will determine whether similar promising findings to ours can be obtained by targeting different α-syn and tau epitopes and assess these ligands by PET imaging. Other synucleinopathies and tauopathies need to be explored as well. In addition, emerging imaging technologies, such as volumetric multispectral optoacoustic tomography, should allow a better penetration depth of the fluorescent brain signal, resulting in its 3D spatial resolution in at least large portions of the brain (*49*, *50*). Overall, these specific anti–α-syn and anti-tau sdAb imaging ligands have great potential as in vivo diagnostic markers for synucleinopathies versus tauopathies, respectively, and sdAb-based imaging may be applicable to a variety of protein conformational disorders.

## MATERIALS AND METHODS

### sdAb generation

The llama immunizations and the generation of phage display libraries of sdAbs (VHHs) were performed using the services of ProSci Inc. (Poway, CA). Bleeds were obtained from the llamas at several intervals during the immunization protocol and screened in the Sigurdsson laboratory for binding to various α-syn and tau preparations, respectively, which guided which bleeds to use to generate the sdAb libraries (fig. S1). See Fig. 1 for a schematic overview of the development of the anti–α-syn and anti-tau sdAb imaging agents.

#### Anti–α-syn sdAbs

More than 50 α-syn sdAb clones were initially identified by panning for rec α-syn in its solution and solid phase. The culture supernatants from these clones were then screened in the Sigurdsson laboratory for binding to various α-syn preparations in ELISAs and to human synucleinopathy brain sections, resulting in the selection of the 2D10 and 2D8 sdAb clones for imaging.

#### Anti-tau sdAbs

Likewise, more than 50 tau sdAb clones were initially identified by panning for rec tau 441 in its solution and solid phase and further screened in the Sigurdsson laboratory for binding to various tau preparations in ELISAs and on tauopathy brain sections, resulting in the selection of the 2B8 sdAb clone for imaging, based primarily on its strong binding to tau lesions on brain sections.

### Immunizations

#### Anti–α-syn sdAbs

A llama was immunized with a rec α-syn (140 amino acids, rPeptide) with seven injections in various adjuvants, as detailed in table S1.

#### Anti-tau sdAbs

A llama was immunized with five injections of the longest isoform of rec tau (441 amino acids, rPeptide) in various adjuvants, followed by two immunizations with PHF-enriched tau in adjuvant, as detailed in table S2. The PHF-tau was extracted from a human tauopathy brain with mixed AD/Pick’s disease pathology [National Disease Research Interchange (NDRI), Philadelphia, PA], as we have described previously (*51*, *52*). In both llamas, bleeds were taken 1 week after boosts to determine antibody response and to obtain PBMCs to generate the phage display libraries of sdAbs detecting α-syn or tau in solid and solution phases.

### Antibody titer

Antibody levels in serum were determined by indirect ELISA using the following protocol. The rec human α-syn antigen (140 amino acids) or 2N4R (441 amino acids) of rec tau was coated onto 96-well Corning 9018 high-binding polystyrene assay plates at 2 μg/ml in phosphate-buffered saline (PBS) at 4°C overnight. The plate was then washed four times with PBS containing 0.05% Tween 20 (PBS-T) and blocked for 2 hours at room temperature with PBS-T containing 5% non-fat dried milk (PBS-T/milk). The plate was then washed again four times with PBS-T. Dilutions of sera were made in PBS-T/milk and incubated in the plate for 1 hour at room temperature followed by four washes in PBS-T. It was then incubated with rabbit anti-llama immunoglobulin G (IgG) (H + L) (Life Technologies) at 1:10,000 dilution in PBS-T/milk for 1 hour at room temperature followed by four washes in PBS-T. The bound antibody was detected with goat anti-rabbit IgG (H + L)–horseradish peroxidase (HRP) (Jackson Laboratories) at 1:10,000 dilution in PBS-T/milk after a 45-min incubation at room temperature.

The serum was also reacted on dot blots to assess the reactivity of the different bleeds for supernatants of brain homogenates from human synucleinopathy brains and PHF-enriched tau derived from homogenates of tauopathy brains, respectively. In the α-syn study, these were cortical brain homogenates from cases that had been diagnosed with LBD and were obtained from the Banner Institute Arizona [provided by T. Beach (*53*)]. These brain samples were selected on the basis of their strong reactivity with commercial α-syn antibodies suggesting extensive Lewy bodies. In the tau study, these were cortical brain homogenates from a mixed AD/Pick’s disease brain (not the same as used for PHF extraction for immunization) and a mixed AD/progressive supranuclear palsy (PSP) brain. Both tauopathy brains were obtained from the NDRI (Philadelphia, PA). Briefly, an LSS of brain homogenates from these human LBD cases or PHF-tau was spotted onto nitrocellulose membranes (1 μl of 1 mg/ml) and then incubated with serum from each of the seven time points, pretreatment through bleed 6, at a 1:1000 dilution overnight. Following this, blots were washed and incubated with HRP-conjugated llama secondary antibody (ProSci Inc., catalog no. 8417-HRP-2) at 1:2000 and developed together for each llama to ensure that time in the developing fluid and exposure would be identical.

### Library construction

Approximately 100 ml of llama blood from each llama was separately collected in EDTA-coated tubes (Becton Dickenson), and PBMCs were isolated using Histopaque-1077 (Sigma-Aldrich). Approximately 1 × 10^8^ PBMCs were isolated from the specific bleeds listed above and RNA was prepared using RNeasy (Qiagen). cDNA was synthesized from a total of 50 μg of RNA (25 μg from each bleed) using random hexanucleotide primers and SuperScript reverse transcriptase (Life Technologies). SdAbs were cloned in a two-step polymerase chain reaction (PCR) process using the cDNA as a template. The first PCR amplified the variable domains of all Ig heavy chains, both single chain (VHH) and conventional antibodies (VH) using CaL1/CaL2 primers: 5′-GTCCTGGCTGCTCTTCTACAAGG-3′/5′-GGTACGTGCTGTTGAACTGTTCC-3′. The second nested PCR specifically amplified llama sdAbs (ProSci proprietary primers). PCR products were gel-purified, and the DNA fragments encoding the VHH domains were cloned into phage display vector pADL-23c (Antibody Design Labs) and electroporated into *Escherichia coli* TG1 cells, yielding a library of approximately 1 × 10^9^ in size.

### Initial selection of sdAbs

Phage displaying sdAbs were rescued from individual libraries with helper phage M13K07 and subjected to biopanning in two different ways: solid-phase and solution-phase panning. For solid-phase panning, rec α-syn or rec tau was adsorbed on 2-μm-diameter polystyrene beads (Polysciences Inc.), blocked with PBS-T containing 5% non-fat dried milk, and incubated with 2 × 10^11^ phage in 1 ml of PBS-T/milk for 1 hour at room temperature. For solution-phase panning, rec α-syn or rec tau were biotinylated using Lightning-Link Rapid Biotin (Innova Biosciences), and 1 μg of biotinylated α-syn or tau was incubated with 2 × 10^11^ phage in 1 ml of PBS-T/milk for 1 hour at room temperature. Polystyrene beads were captured by centrifugation and washed extensively with PBS-T. Biotinylated α-syn or tau bound to phage was captured with streptavidin (SA)–coated magnetic Dynabeads (Life Technologies) and extensively washed with PBS-T. For both the solid- and solution-phase approaches, bound phage was eluted with both high pH (100 mM triethylamine, pH ~10) and low pH (100 mM glycine, pH ~2.5) for 5 min and neutralized with 1 M tris (pH 7.5). *E. coli* strains TG1 and SS320 were then infected with eluted phage and used for a subsequent round of panning (TG1) or to express antibodies (SS320).

### Bacterial expression of sdAbs for screening

Individual SS320 clones were grown in a 96-well plate format in 100 μl of 2-YT broth/1% glucose/ampicillin (100 μg/ml) overnight at 37°C. Ten microliters of the overnight cultures was inoculated into 1 ml each of 2-YT/0.1% glucose/ampicillin (100 μg/ml) in deep 96-well blocks and grown at 37°C at 200 rpm for 3 hours until cells were in log phase. SdAb expression was then induced with isopropyl-β-d-thiogalactopyranoside (IPTG; 1 mM final concentration), and the temperature was reduced to 30°C overnight. The next day, bacterial cultures were centrifuged at 3200*g* for 10 min and the supernatant was removed. The remaining bacterial pellets were frozen at −70°C. Bacterial pellets were then thawed, resuspended in 300 μl of PBS, and incubated for 30 min at room temperature. Cellular debris was removed by centrifugation at 3200*g* for 10 min and the antibody-containing supernatants were transferred to a fresh 96-well plate for storage at −70°C until used. ELISA screening of the supernatants was performed using the following protocol. The antigen was coated at 2 μg/ml in PBS at 4°C overnight in 96-well Corning 9018 high-binding polystyrene assay plates. The plates were then washed four times with PBS-T, blocked for 2 hours with PBS-T/5% milk at room temperature, and washed again four times with PBS-T. Subsequently, dilutions of sdAb supernatants (1:1) in PBS-T/5% milk were added for 1 hour at room temperature, and the plates were washed four times with PBS-T, followed by incubation with ProSci’s anti–c-myc-tag antibody (catalog no. PM-7669), 1:1000 in PBS-T/5% milk for 1 hour at room temperature. After four washes in PBS-T, binding was detected with goat anti-mouse IgG-HRP (Jackson Laboratories, catalog no. 115-035-164), 1:5000 in PBS-T/5% milk, incubated 30 to 45 min at room temperature, followed by four washes in PBS-T and color development for 10 min to 1 hour depending on the intensity of the signal. Subsequent screening of high binding supernatants was performed on various α-syn preparations (rec α-syn and homogenate fractions from synucleinopathy brains) or tau preparations (rec soluble and fibrillar, and enriched fractions from human and mouse tauopathy brains). Six clones representing different binding profiles within each cohort were then purified and analyzed further, resulting in the selection of the α-syn sdAb clones, 2D10 and 2D8, and the tau sdAb clone 2B8 for IVIS imaging.

### Purification of sdAbs from bacterial culture

Individual sdAbs or polyclonal sdAbs were purified from 50-ml cultures as follows. Selected clones or the enriched mixture of clones from the specific pannings (for polyclonals) were inoculated into 3 ml of SS320 cells in 2-YT/1% glucose/ampicillin (100 μg/ml) and grown overnight at 37°C at 200 rpm. The next day, 500 μl of this overnight culture was added to 50 ml of 2-YT/0.1% glucose/ampicillin (100 μg/ml) and grown at 37°C at 200 rpm. After optical density at 600 nm (OD_600_) reached 0.7, the culture was induced with IPTG (1 mM final concentration) and grown overnight at 30°C. The next day, the bacteria were collected by centrifugation for 15 min at 3500*g* at room temperature, followed by resuspending the cell pellet in 2.5 ml of ice-cold TES (20 mM tris, 0.5 mM EDTA, and 17% sucrose) and incubation for 1 hour on ice. Then, 5 ml of TES/4 (TES diluted 1:4 in water) was added, and the mixture was incubated on ice for 45 min with occasional mixing. The suspension was then centrifuged at 10,000*g* for 30 min at 4°C, and the supernatant was collected. The his-tagged sdABs were then purified from it by using immobilized metal affinity chromatography, according to the manufacturer’s instructions (Qiagen).

### Mammalian expression and purification of sdAbs

For the mouse imaging studies, the sdAb clones were expressed in a mammalian system to avoid having to endotoxin-purify clones expressed in a bacterial system. pVRC8400-sdAb constructs were made by inserting their individual gene sequences between the 5′ Eco RI and 3′ Afe I sites with a signal peptide, mouse interleukin-2 leader sequence (MYRMQLLSCIALSLALVT) at the N terminus and myc-his tags at the C terminus for detection and purification. The sdAbs were then expressed in Freestyle 293F cells (Invitrogen, catalog no. R790–07). Briefly, Freestyle 293F cells were transiently transfected with the mixture of DNA plasmid and polycation polyethylenimine (PEI; 25 kDa linear PEI, Polysciences Inc., catalog no. 23966). The transfected cells were incubated at 125 rpm in Freestyle 293 expression medium for suspension culture at 37°C with 5% CO_2_. The supernatants were harvested 5 days after transfection and filtered through a 0.45-μm filter, followed by sdAb purification using Ni–nitrilotriacetic acid (NTA) columns (GE Healthcare). The wash buffer consisted of 20 mM Na_3_PO_4_, 100 mM NaCl, and 20 mM imidazole (pH 7.4). The elution buffer consisted of 20 mM Na_3_PO_4_, 100 mM NaCl, and 500 mM imidazole (pH 7.4). Following elution, each sdAb was dialyzed into PBS and its concentration was determined by a bicinchoninic acid (BCA) assay.

### Preparation of recombinant p-tau

Recombinant p-tau was generated as described previously (*54*). Briefly, the 1N4R isoform of tau was expressed along with GSK-3β via the PIMAX approach (*55*). The BL21 Codon plus strain containing the p-tau–expressing plasmid was grown at 37°C until OD_600_ of 0.3 to 0.5 before 2 hours of induction with 0.5 mM IPTG. The cell pellet was then suspended in purification buffer [20 mM tris-HCl (pH 5.8), 100 mM NaCl, 1 mM phenylmethylsulfonyl fluoride (PMSF), and 0.2 mM orthovanadate] and treated with lysozyme (1 mg/ml) at 30°C for 30 min. The mixture was then sonicated (Branson Digital Sonifier 450; 30% amplitude; total process time, 3 min; pulse-ON time, 5 s; pulse-OFF time, 5 s) and centrifuged at 17,000*g* for 40 min at 4°C. Subsequently, the supernatant was placed in a boiling water bath for 30 min and then on ice for 30 min with occasional gentle shaking. It was then centrifuged at 17,000*g* for 50 min at 4°C and transferred to another tube and supplied with a final concentration of 0.5 mM dithiothreitol and 1 mM EDTA. One OD_280_ of purified recombinant Tobacco Etch Virus (TEV) protease was subsequently added to digest each 100 OD_280_ of the sample at 4°C overnight. The digestion mixture was then centrifuged again at 17,000*g* for 30 min at 4°C. The supernatant with p-tau was collected and concentrated by centrifugation through a spin column (Amicon Centrifugal Filter Unit, Ultra-15, 10K). A gel filtration buffer [20 mM tris-HCl (pH 7.4) and 100 mM NaCl] was added at the end of the concentration step. The centrifugation and buffer change were repeated twice. The final gel filtration buffer–equilibrated solution was injected to a Superdex 200 10/300 GL column (GE Healthcare Life Sciences, USA). Size exclusion chromatography was done on an AKTA explorer fast protein liquid chromatography unit at 4°C under a flow rate of 0.3 ml/min. Fractions containing p-tau were pooled and concentrated by a spin column. After the concentration was completed, the protein solution was collected and supplemented with 10% glycerol (v/v) before storage at −80°C.

### Specificity of the sdAbs against α-syn and tau pathology and epitope mapping of the antibodies against α-syn and tau peptide libraries

#### Anti–α-syn sdAb

The 2D10 and 2D8 sdAbs were reacted on dot blots with various human and mouse brain samples to assess their specificity and with an α-syn peptide library (synthesized by Genscript Inc., Paramus, NJ) to determine their binding epitopes. The library covered all 140 amino acids of α-syn, with peptides 1 to 13 consisting of 15 amino acids and peptide 14 consisting of 10 amino acids (table S3). Peptide-peptide overlaps were five amino acids.

#### Anti-tau sdAb

The 2B8 sdAb was reacted on a dot blot with various human and mouse brain samples to assess their specificity and with a tau peptide library (synthesized by Genscript Inc., Paramus, NJ) to determine its binding epitope. The library covered all 441 amino acids of the longest isoform of the tau protein, with peptides 1 to 24 consisting of 25 amino acids and peptide 25 consisting of 9 amino acids (table S4). Peptide-peptide overlaps were seven amino acids.

The human and mouse brain samples used for dot blots consisted of the LSSs of these tissues described in the “Brain homogenization, preparation of α-syn and tau fractions, and Western blots” section below, or the PHF-tau enriched from human tauopathy brains (see the “Isolation of PHF tau” section below). Their protein concentrations were measured and normalized to 1 mg/ml in homogenization buffer, with 5 μg of each sample dot-blotted onto nitrocellulose membrane (0.2 μm) and air-dried for 30 min. For the epitope mapping, peptides were dissolved into a small amount of dimethyl sulfoxide (DMSO) and subsequently diluted into PBS at a concentration of 1 mg/ml, with 5 μg of each peptide dot-blotted onto nitrocellulose membrane and air-dried for 30 min.

After blocking the membrane for 1 hour with 5% milk in 0.1% Tween-20 in tris-buffered saline (TBS-T), the blots were incubated overnight in a cold room with either 2D8 or 2D10 for α-syn and 2B8 for tau (containing his tag, 0.01 mg/ml) in Superblock (Thermo Fisher Scientific). Following several washes in TBS-T, the blots were then incubated for 1 hour at room temperature with anti-6× His tag antibody (1:2000, ABCAM, EPR20547) in Superblock, washed with TBS-T, and the signal was detected with IRDye 800CW secondary antibody (1:10,000, LI-COR Biosciences). Images of immunoreactive bands were quantified by LI-COR Image studio lite 5.2.

### BLI binding affinity assay

All the BLI experiments were performed on a ForteBio Octet RED96 instrument. Experiments were conducted at room temperature in a 96-well black flat-bottom plate with shaking speed at 1000 rpm. All samples were dissolved in 200 μl of the assay buffer of 1× PBS and 0.05% Tween-20 at pH 7.4.

#### Solid phase

An SA biosensor was used to measure the biophysical interaction between the sdAbs and the biotinylated α-syn preparation (rec α-syn, human α-syn insoluble P1 fraction, or tg M83 mouse human α-syn insoluble SP fraction) or tau preparation (rec tau or rec p-tau), each of which was labeled with biotin using the EZ-Link NHS-PEG_4_-Biotin. First, 1 mg of protein was dissolved in 0.5 ml of 1× PBS (pH 7.4). Immediately before use, a 10 mM solution of the biotin reagent EZ-LinkTM NHS-PEG_4_-Biotin was prepared in DMSO and then added to the protein solution and rotated for 30 min at room temperature, followed by removal of excess biotin by dialysis. Before the initiation of BLI, the SA biosensor was hydrated in the assay buffer for 10 min, and then a baseline was established in the assay buffer for 120 s, followed by loading of optimized concentration of biotin-labeled α-syn preparation for 120 s (typically 400 nM for rec α-syn). Similar optimized concentrations of the tau preparations were used. Subsequent steps included generating another baseline in the assay buffer for 120 s, then association for 300 s at different concentrations of sdAb, and dissociation in the assay buffer for 400 s. A reference biosensor was loaded with the biotinylated α-syn or tau preparations and run with an assay buffer blank for the association and dissociation steps. The biosensor tips were regenerated by cycling them three times for 10 s each between 10 mM glycine (pH 2) and assay buffer.

#### Solution phase

Ni-NTA biosensor was used to measure the biophysical interaction between the his-tagged sdAbs and the different α-syn preparations [rec-α-syn, human α-syn soluble S1 fraction, or soluble α-syn (LSS—Tg M83 mouse brain fraction] or tau preparations (rec tau or rec p-tau). The Ni-NTA biosensor is preimmobilized with nickel-charged tris-NTA groups for the capture of his-tagged molecules. Before the initiation of BLI, the sensor was hydrated in the assay buffer for 10 min. Then, a baseline was established in the assay buffer for 120 s. Subsequently, the Ni-NTA tips were loaded with his-tagged sdAb at 5 μg/ml for 120 s, resulting in specific interaction between his tag and nickel ions. Then, another baseline step was performed in the assay buffer for 120 s. Subsequently, ligand-loaded biosensor tips were dipped into the solution of the α-syn or tau preparation for 300 s at different concentrations in 1× PBS (pH 7.4) with 0.05% Tween-20 (PBS-T). Last, dissociation was conducted in the assay buffer for 400 s. A reference biosensor was loaded with the his-tagged sdAb and run with an assay buffer blank for the association and dissociation steps. The biosensor tips were regenerated by cycling them three times for 5 s each between 10 mM glycine (pH 2) and assay buffer, followed by recharging for 1 min with 10 mM NiCl_2_.

Data analysis was performed using Data Analysis 11.0 software following reference subtraction using a 1:1 binding model. In a 1:1 bimolecular interaction, both the association and dissociation phases display a time-resolved signal that is described by a single exponential function. Analyte molecules bind at the same rate to every ligand binding site. The association curve follows a characteristic hyperbolic binding profile, with exponential increase in signal followed by a leveling off to plateau as the binding reaches equilibrium. The dissociation curve follows single exponential decay with signal eventually returning to baseline. The full fitting solution for a 1:1 binding is

Association phase:

$$y=R_{\max}\;\frac{1}{1+\frac{k_{d}}{k_{a}*[\mathrm{Analyte}]}}\;\{1-e^{-(k_{a}*[\mathrm{Analyte}]+k_{d})x}\}$$

Dissociation phase:

$$\begin{array}{c} y=y_{0}e^{-k_{d}(x-x_{0})}\\ y_{0}=R_{\max}\;\frac{1}{1+\frac{k_{d}}{k_{a}*[\mathrm{Analyte}]}}\;\{1-e^{-(k_{a}*[\mathrm{Analyte}]+k_{d})x_{0}}\} \end{array}$$

The curve fit algorithm is based on the Levenberg-Marquardt fitting routine to find the best curve fit. In the 1:1 model, the data fit into the equations above as follows: *x* = time, *y* = nm shift, Analyte = concentration. The algorithm is used to fit for *R*_max_, *k*_d_ and *k*_a_ values, and *K*_D_ is calculated by dividing *k*_d_ by *k*_a_ (i.e., *K*_D_ = *k*_d_/*k*_a_).

According to the ForteBio analytical protocol, when running a full kinetic profile with several analyte concentrations, the data are analyzed globally by fitting both association and dissociation phases for several analyte concentrations simultaneously using the same set of rate constants with a 1:1 binding model using the global fitting function (grouped by color, *R*_max_ unlinked by sensor). Global analysis of a wide range of analyte concentrations provides robust analysis and accurate estimation of binding constants. See the company’s website (www.sartorius.com) for a detailed application note on biomolecular binding kinetics assays on the Octet Platform.

### Mouse models, injections of labeled antibodies, and IVIS imaging

All mouse experiments were performed under an Institutional Animal Care and Use Committee–approved protocol with the mice housed in Association for Assessment and Accreditation of Laboratory Animal Care–approved facilities with access to food and water ad libitum.

#### M83 mice

The homozygous M83 mice express the A53T mutation of human α-syn via the mouse prion promoter at about 12 times the level of endogenous mouse α-syn. This model develops α-syn inclusions with age that are extensive in the homozygous mice at the age used here (5 to 8 months), and various behavioral deficits have been reported in this model (*20*).

#### PS19 mice

The heterozygous PS19 mice express the P301S mutation of human tau 1N4R driven by the mouse prion promoter at about five times the level of endogenous mouse tau (*56*). The transgene is expressed throughout the brain with the most severe tau pathology typically seen in the brainstem, but extensive tau pathology is seen throughout the brain, including the cortex. The distribution of tau pathology is comparable to the JNPL3 model as they use the same promoter. These lesions have been reported to be associated with behavioral deficits affecting cognition and motor function. In our colonies, the heterozygous PS19 mice develop brain tau aggregates at an earlier age than the homozygous JNPL3 mice, presumably because of greater transgene expression. However, at these ages, the homozygous JNPL3 mice are expected to have substantially more tau deposits than the heterozygous PS19 mice.

#### JNPL3 mice

The homozygous JNPL3 mice express the P301L mutation of human tau 0N4R driven by the mouse prion promoter at approximately twice the level of endogenous mouse tau (*57*). The transgene is expressed throughout the brain with the most severe tau pathology typically seen in the brainstem, but extensive tau pathology is seen throughout the brain, including the cortex. When this model was first described, most of the homozygous mice developed severe motor deficits by 10 months, which is likely related to tau pathology in the brainstem and spinal cord. These functional impairments are now often delayed by a few months in females and are often not seen in males, even at an old age.

#### Anti–α-syn sdAbs

Imaging using IVIS (Lumina XR, PerkinElmer) of α-syn lesions was performed in four different mouse models: homozygous M83 α-syn A53T mice (14 females and 7 males, 5 to 8 months; JAX no. 004479), WT control mice of the same strain background as the M83 model (2 females and 2 males, 6 to 8 months), heterozygous PS19 tau P301S mice as cross-reactivity control (2 females and 2 males, 7 to 10 months; JAX no. 008169), and WT control mice of the same strain background as the PS19 model (2 females and 2 males, 7 to 10 months). See table S5.

#### Anti-tau sdAb

Imaging using IVIS (Lumina XR, PerkinElmer) was conducted in five different mouse models: homozygous JNPL3 mice (8 males, 17 months; Taconic 2508), heterozygous PS19 mice (3 males, 6 to 9 months; JAX no. 008169), WT control mice of the same strain background as JNPL3 mice (3 females, 15 to 18 months), WT controls of the same strain background as PS19 mice (2 males, 9 to 14 months), and homozygous M83 A53T tg α-syn (JAX no. 004479) mice (6 males, 6 to 7 months) as cross-reactivity controls. See table S6.

Right before IVIS imaging, the mice were anesthetized with 2% isoflurane, maintained with 1.5% isoflurane in 30% O_2_, and then injected intravenously into the femoral vein with VivoTag 680XL (PerkinElmer)–labeled sdAb (10 mg/kg), as described previously in detail for other substances (*18*). The fur on the head was always shaved off, typically on the body, except that it is not necessary in albinos, to eliminate nonspecific light diffraction from the fur.

Immediately following the intravenous sdAb injection, the mice were imaged at defined intervals as detailed in Figs. 4 and 6 and figs. S13 and S14. The animals were kept anesthetized within the imaging chamber with 2% isoflurane delivered with O_2_ via a nosecone at a flow rate of 0.4 liters/min. Their body temperature was maintained at 37°C via the thermoelectrically controlled imaging stage. Using Living Image software (PerkinElmer), fluorescent images were obtained with the following settings: mode, fluorescent; illumination, epi-illumination; exposure time and binning, auto (30 s under these conditions); excitation filter, 675 nm; and emission filter, Cy5.5 (695 to 770 nm). To facilitate analysis, the image data were automatically time- and date-stamped.

#### Analysis of IVIS images

Images were analyzed using Living Image software from PerkinElmer. First, images of each mouse from different time points were loaded together as a sequence. Next, this group of images was set to the same color scale to facilitate visual comparison, and circular ROI was drawn in the brain region of each animal. Subsequently, the calibrated unit of radiant efficiency (photons/s/cm^2^/Str/mW/cm^2^) was reported for each ROI, and a table with all ROI values in the sequence was created, saved, and used for graphing the IVIS imaging profile of that mouse.

### Human brain tissue

For the anti–α-syn sdAb characterization, frozen and fixed brain samples from the temporal cortex were obtained from subjects with LBD from the Arizona Study of Aging and Neurodegenerative Disorders and Brain and Body Donation Program of Banner Sun Health Research Institute in Sun City, AZ (https://brainandbodydonationregistration.org/) (*53*). Fixed cortical brain samples from a subject with extensive cortical α-syn inclusions were also used for the initial characterization of the sdAbs (NDRI, Philadelphia, PA).

For the anti-tau sdAb characterization, several tauopathy brains with AD pathology (tau and Aβ lesions) or mixed AD and PSP or AD and Pick’s disease tau pathologies were obtained from the NDRI (Philadelphia, PA). Typically, one of its hemispheres was formalin-fixed and the other one was frozen, and samples from the frontal cortex were processed and analyzed.

### Immunohistochemistry of brains

For sdAb characterization, the paraffin-embedded fixed human brain tissue was sectioned at 10 μm, mounted onto slides, deparaffinized, defatted, stained with the different sdAbs using standard procedures, and detected with rabbit-anti llama secondary antibody (ProSci Inc.), followed by incubation with anti-rabbit antibody, avidin, and peroxidase (Vector Labs kit) and reaction with diaminobenzidine (DAB)/Ni/peroxide, as described previously (*58*). After imaging, the mouse brains were processed as previously described in detail (*59*). Briefly, the mice were perfused trans-aortically with PBS, their brains were then removed, and the right hemisphere, with a sliver of the left hemisphere to maintain brain integrity, was immersion-fixed in 2% paraformaldehyde/lysine/periodate overnight. The left hemisphere was snap-frozen on CO_2_ blocks and stored at −80°C until processed for Western blots (see below). Following the overnight fixation, the right hemisphere was placed in 2% DMSO in 20% glycerol phosphate buffer overnight or longer at 4°C and then sectioned coronally into serial 40-μm sections to detect 680XL-sdAb signal and to determine its subcellular location by costaining with markers of α-syn protein and the endosomal-autophagosomal-lysosomal system. Sectioned series (400 μm apart) were stored in ethylene glycol cryoprotectant at −30°C until used for immunohistochemistry.

Immunofluorescence staining was performed per standard protocol on free-floating sections. Briefly, after PBS washes, 0.3% Triton X-100 permeabilization, and block in 5% BSA, tissue was incubated with antibodies (1:500 to 1:1000) overnight at 4°C [α-syn (α-syn 211, α-syn pS129), tau (PHF1 and MC1), early endosomes (EEA1), late endosomes/lysosomes (rab7), and autophagosomes (LC3)]. Bound antibodies were detected with an Alexa Fluor 488 goat anti-mouse/rabbit IgG (Invitrogen) and nuclei with 4′,6-diamidino-2-phenylindole. After coverslipping with ProLong Gold, the tissue was analyzed with an LSM 800 Zeiss confocal laser scanning microscope (Axioimager).

### Confocal imaging analysis

The extent of staining with each label and their colocalization was measured by calculating the Manders’ correlation coefficient using the JACop plugin in ImageJ software (*60*). Manders’ coefficients represent the proportion of confocal fluorescence signals, where 0 is defined as no colocalization and 1 is defined as complete colocalization. The Manders’ coefficients in Figs. 8 to 10 were calculated in the merged layers shown in the third image from the left. These coefficients imply the amount of labeled 2D10 and 2D8 overlapping with α-syn 211, α-syn PS129, EEA1, Rab7, and LC3 or labeled 2B8 overlapping with PHF1, MC1, EEA1, and Rab7. Confocal Z-stack images were 3D reconstructed by the ZEISS ZEN 3.4 imaging software. This analysis was performed to determine (i) the residual signal of the injected fluorescently labeled sdAb, (ii) the extent of α-syn or tau pathology in the brain, (iii) the degree of colocalization of injected sdAb signal with α-syn or tau antibody staining, or (iv) the extent of sdAb presence with endosomes, autophagosomes, and lysosomes.

### Brain homogenization, preparation of α-syn and tau fractions, and Western blots

The left hemisphere of the mouse brains was homogenized in (5 × v/w) modified radioimmunoprecipitation assay buffer [50 mM tris-HCl, 150 mM NaCl, 1 mM EDTA, and 1% Nonidet P-40 (pH 7.4)] with protease and phosphatase inhibitors [1× of protease inhibitor mixture (cOmplete, Roche), 1 mM NaF, 1 mM Na_3_VO_4_, 1 nM PMSF, and 0.25% sodium deoxycholate] and kept on ice briefly until centrifuged to retrieve the supernatant. The homogenate was centrifuged at 20,000*g* for 20 min at 20°C, and the supernatant was collected as soluble α-syn or tau fraction [low speed supernatant (LSS)]. The protein concentration of each sample was measured by BCA assay and the samples were adjusted to the same concentration with the homogenization buffer with 5 μg of protein loaded per well. To obtain the sarkosyl insoluble fraction, equal amounts (2 mg) of protein from the LSS were mixed with 10% sarkosyl in phosphate buffer to a final 1% sarkosyl concentration. Each sample was then mixed for 30 min at room temperature in a Mini LabRoller (Labnet H5500), followed by ultracentrifugation at 100,000*g* for 1 hour at 20°C in a Beckman Coulter Optima MAX-XP Ultracentrifuge. Subsequently, each pellet was washed with 1% sarkosyl solution and centrifuged again at 100,000*g* for 1 hour at 20°C. The SP fraction was then air-dried for 30 min and mixed with 50 μl of modified O+ buffer (62.5 mM tris-HCl, 10% glycerol, 5% β-mercaptoethanol, 2.3% SDS, 1 mM EDTA, 1 mM EGTA, 1 mM NaF, 1 mM Na_3_VO_4_, 1 nM PMSF, and 1× of the protease inhibitor mixture). Likewise, the LSS was eluted in the same manner with O+ buffer (1:5). All of the samples were boiled and 10 μl of each sample was electrophoresed on 12% SDS–polyacrylamide gel electrophoresis gels and transferred to nitrocellulose membranes.

Cortical samples from the human LBD brains were homogenized in TBS and were centrifuged at 3000*g* for 20 min. The supernatant was retained as the total soluble fraction (S1), while the pellet was resuspended in tris-EDTA buffer and labeled as the total insoluble fraction containing the larger α-syn aggregates (P1).

Following protein transfer from gel to membrane, all blots for α-syn detection were treated for 30 min with 4% paraformaldehyde to prevent α-syn from washing off the membrane (*61*). The blots were blocked in Superblock (Thermo Fisher Scientific) and incubated with (i) anti–α-syn sdAb/syn 211 (1:1000), PA5-13401 (1:1000), and glyceraldehyde-3-phosphate dehydrogenase (GAPDH, 1:4000; Cell Signaling Technology, D16H11) and (ii) anti-tau sdAb/PHF1 (1:1000, gift from P. Davies), CP27 (1:500, gift from P. Davies), and GAPDH (1:4000; Cell Signaling Technology, D16H11). The blots were then incubated with either IRDye 800CW or IRDye 680RD secondary antibodies (1:10,000; LI-COR Biosciences). Images of immunoreactive bands were quantified by LI-COR Image studio lite 5.2.

### Isolation of PHF tau

PHF-enriched tau was isolated from human tissue obtained from patients with mixed pathology of AD and PSP as well as AD and Pick’s disease using methods described previously (*51*, *52*). Brain tissue was homogenized in buffer containing 0.75 M NaCl, 1 mM EGTA, 0.5 mM MgSO_4_, and 100 mM 2-(*N*-morpholino) ethanesulfonic acid (pH 6.5) and centrifuged at 11,000*g* for 20 min. The resulting LSS was then incubated with 1% sarkosyl for 1 hour at room temperature. This mixture was centrifuged at 100,000*g* for 60 min. The supernatant was removed and the pellet was washed with 1% sarkosyl. The tau was then resolubilized by being briefly heated to 37°C in 50 mM tris-HCl buffer and then dialyzed in PBS overnight.

### Statistics

Correlation analysis between the different parameters described in Figs. 5 and 7 was analyzed by Pearson correlation after verifying that the data followed normal distribution (Prism 9.0; GraphPad). For correlations between IVIS signal and insoluble and soluble α-syn or insoluble and soluble tau, all of the sdAb-injected mice were included (tables S5 and S6). For the confocal colocalization analysis, Manders’ correlation coefficients were measured (JACop plugin, ImageJ).

*Note added in proof*: After the manuscript was accepted for publication, the authors became aware of four additional papers that reported α-syn sdAbs but none have been examined as in vivo diagnostic imaging probes following an intravenous injection. These additional papers are:

Mahajan *et al.* Computational affinity maturation of camelid single-domain intrabodies against the nonamyloid component of alpha-synuclein, *Scientific Reports*, **8**, 2018. 10.1038/s41598-018-35464-7.

Butler* et al.* α-Synuclein fibril-specific nanobody reduces prion-like α-synuclein spreading in mice, *Nature Communications*, **13**, 2022. 10.1038/s41467-022-31787-2.

Kulenkampff *et al.* An antibody scanning method for the detection of α-synuclein oligomers in the serum of Parkinson's disease patients, *Chemical Science*, **13**, 2022. 10.1039/D2SC00066K.

Hmila *et al*. Novel engineered nanobodies specific for N‐terminal region of alpha‐synuclein recognize Lewy‐body pathology and inhibit in-vitro seeded aggregation and toxicity, *The FEBS Journal*, **289**, 2022. 10.1111/febs.16376.

## Notes

View/request a protocol for this paper from *Bio-protocol*.
