# Supplementary material for: Single-domain antibody–based noninvasive in vivo imaging of α-synuclein or tau pathology
Source: Sci Adv. 2023 May 10;9(19):eadf3775. doi: 10.1126/sciadv.adf3775 (PMC10171817; doi:10.1126/sciadv.adf3775)
Supplement: Supplementary file 1 — Figs. S1 to S22 Tables S1 to S8 [file sciadv.adf3775_sm.pdf]

Science Advances
AAAS

Supplementary Materials for

Single-domain antibody–based noninvasive in vivo imaging of α-synuclein or tau pathology

Yixiang Jiang *et al.*

Corresponding author: Einar M. Sigurdsson, einar.sigurdsson@nyulangone.org

Sci. Adv. **9**, eadf3775 (2023)
DOI: 10.1126/sciadv.adf3775

This PDF file includes:

Figs. S1 to S22
Tables S1 to S8

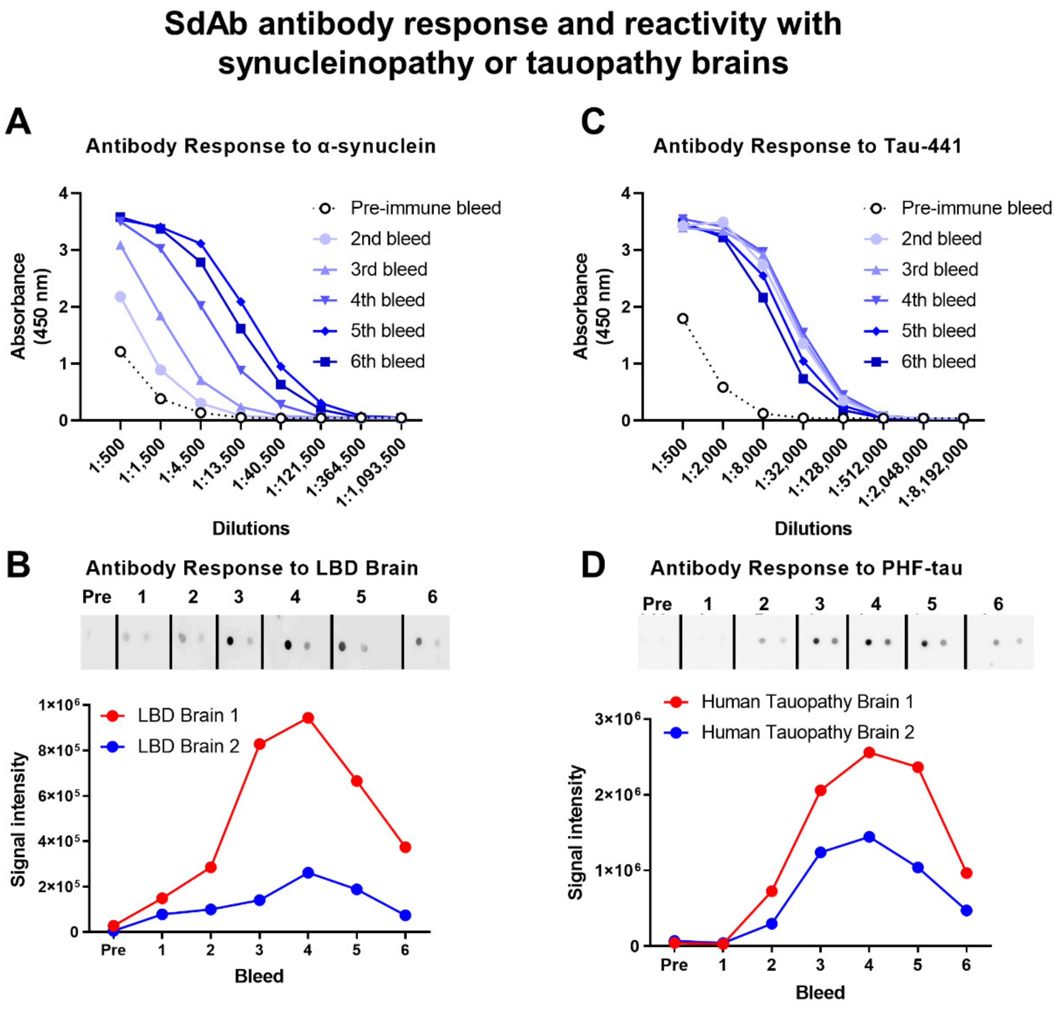

Fig. S1: SdAb antibody response and reactivity with synucleinopathy and tauopathy brains.

(A) SdAb titer against rec α-syn in the α-syn immunized llama peaks at bleed 5 and remains stable through bleed 6. For immunization schedule, see **Table S1**. Some auto-sdAbs against rec α-syn were detected in the pre-bleed but a strong sdAb response against rec α-syn was detected in bleed 2 and onwards. **(B)** SdAbs in bleeds from an α-syn immunized llama show strong immunoreactivity with brain homogenates from cases with Lewy body dementia (LBD). For both cases, signal was strongest in bleed 4.

(C) SdAb titer against rec tau in the tau immunized llama peaks at bleed 2 and remains stable through bleed 6. For immunization schedule, see **Table S2**. Some auto-sdAbs against tau were detected in the pre-bleed but a strong sdAb response against tau was detected in bleed 2 and onwards. **(D)** Bleed 4 shows strongest reactivity towards paired helical filament (PHF)-enriched tau derived from human tauopathy brains.

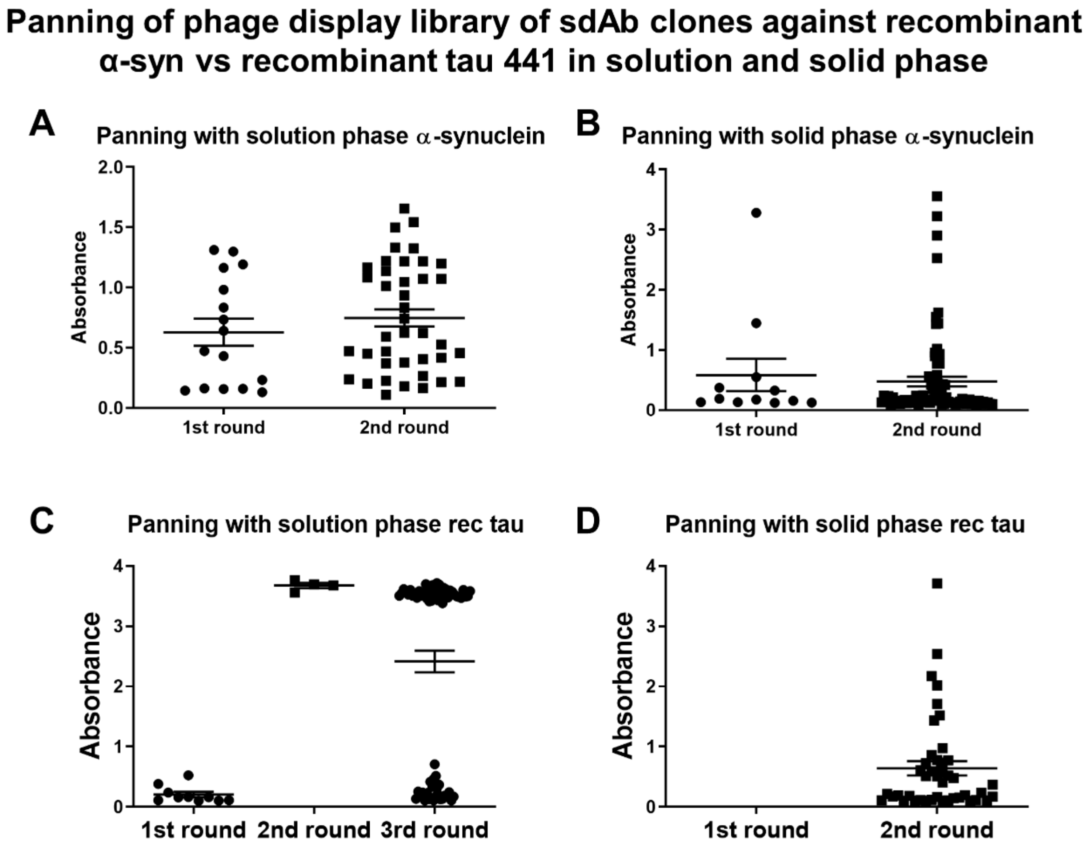

Fig. S2: Phage display library panning against recombinant human α-syn (rec α-syn) in solution and solid phase (A-B), and against recombinant longest isoform (2N4R) of human tau (rec tau) in solution and solid phase (C-D).

Phage display library panning against (**A**) biotinylated recombinant human α-syn (solution phase) resulted in enrichment of positive clones (Abs >0.1) with 9% (first round: two 96 well plates) and 48% (second round – one plate) positivity. (**B**) Panning against solid phase recombinant human α-syn resulted in 6% (1st round) and 43% (2nd round, 2 plates) positivity. Phage display library panning against (**C**) biotinylated- (solution phase) and (**D**) solid phase rec tau resulted in enrichment between rounds of positive clones (Abs > 0.1) with 85% (3rd round: one plate) and 24% (2nd round – 2 plates) positivity, respectively.

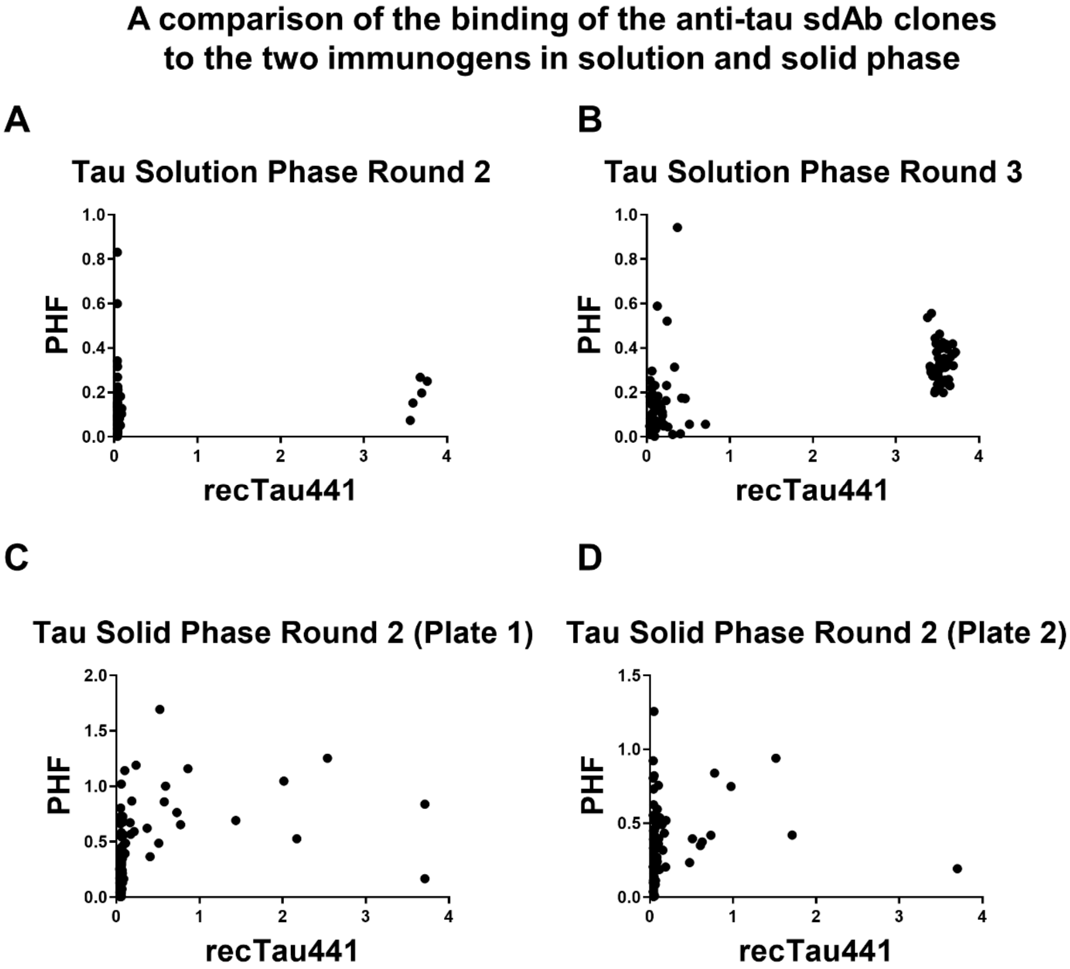

Fig. S3: A comparison of the binding of the anti-tau sdAb clones to the two immunogens in solution and solid phase.

(A-D) Some sdAbs preferably bind to the same paired helical filament (PHF) enriched fraction that was used for immunizations 6-7 (derived from mixed Alzheimer's/Pick's disease brain), whereas others bind better to recombinant longest isoform (2N4R) of human tau (rec tau) or bind well to both PHF and rec tau. Note that these are culture supernatants and clone expression may vary which will affect absorbance values. Supernatants can be expected to contain 0.1-1 μg/mL.

Specificity of sdAb clones for human synucleinopathy vs tauopathy

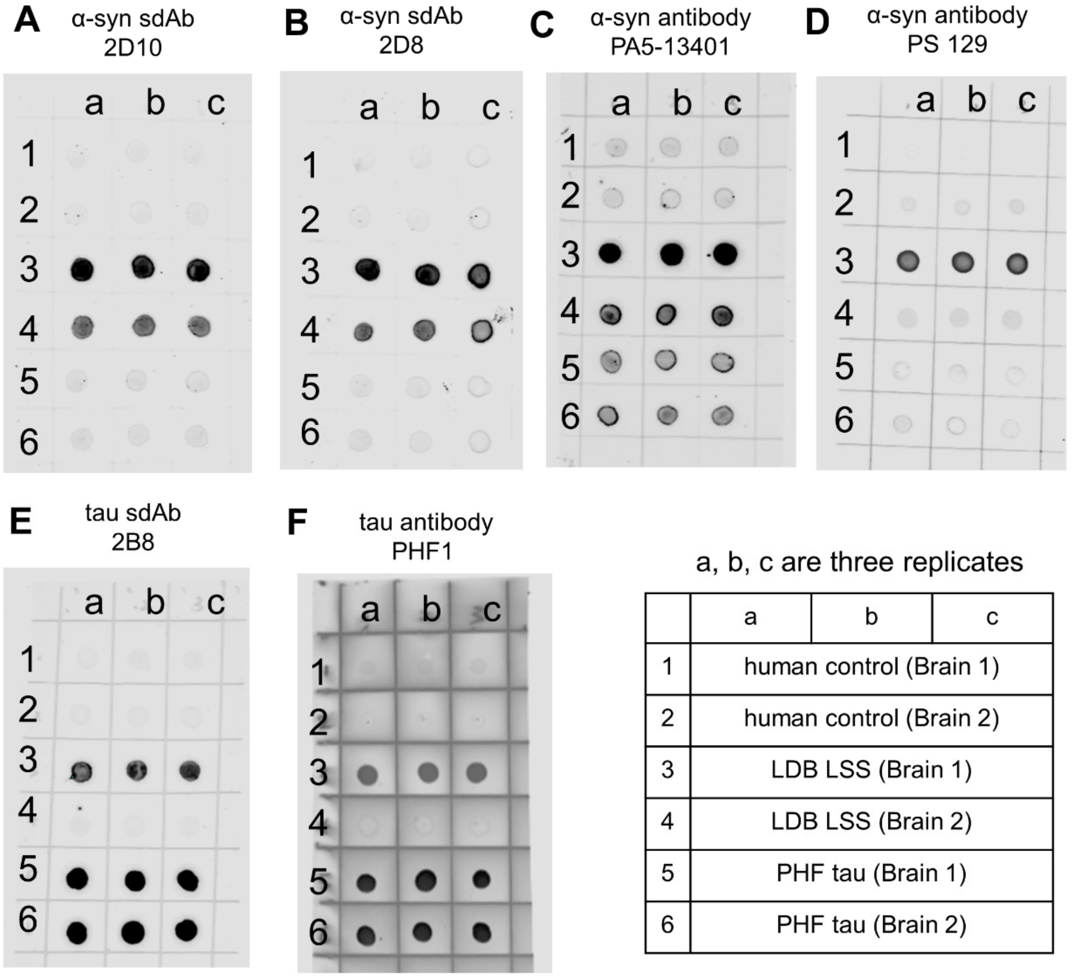

	a	b	c
1	human control (Brain 1)		
2	human control (Brain 2)		
3	LDB LSS (Brain 1)		
4	LDB LSS (Brain 2)		
5	PHF tau (Brain 1)		
6	PHF tau (Brain 2)		

Fig. S4: Reactivity of anti-α-syn sdAbs and anti-tau sdAb to LBD brain tissue or to pathological PHF tau, compared to control brain tissue.

The sdAb antibodies and positive control antibodies were reacted with brain homogenate supernatant from two control brains (lanes 1-2), two LBD brains (lanes 3-4), and PHF isolated from two tauopathy brains (lanes 5-6). Each sample was blotted in triplicates (a-c). The anti-α-syn sdAbs 2D10 and 2D8 are specific for synucleinopathy (A-B) and the anti-tau sdAb 2B8 is specific for tauopathy (E). The PHF material likely contains some α-syn aggregates as per reaction with positive control anti-αsyn antibody PA5-13401 (C, lanes 5 and 6). In addition, 2D10 and 2D8 appear to be more sensitive to detect synucleinopathy than positive control p-S129 antibody (compare lanes 4 in A, B and D). The positive control tau antibody, PHF1, has similar reactivity profile as the sdAb 2B8 (compare E and F).

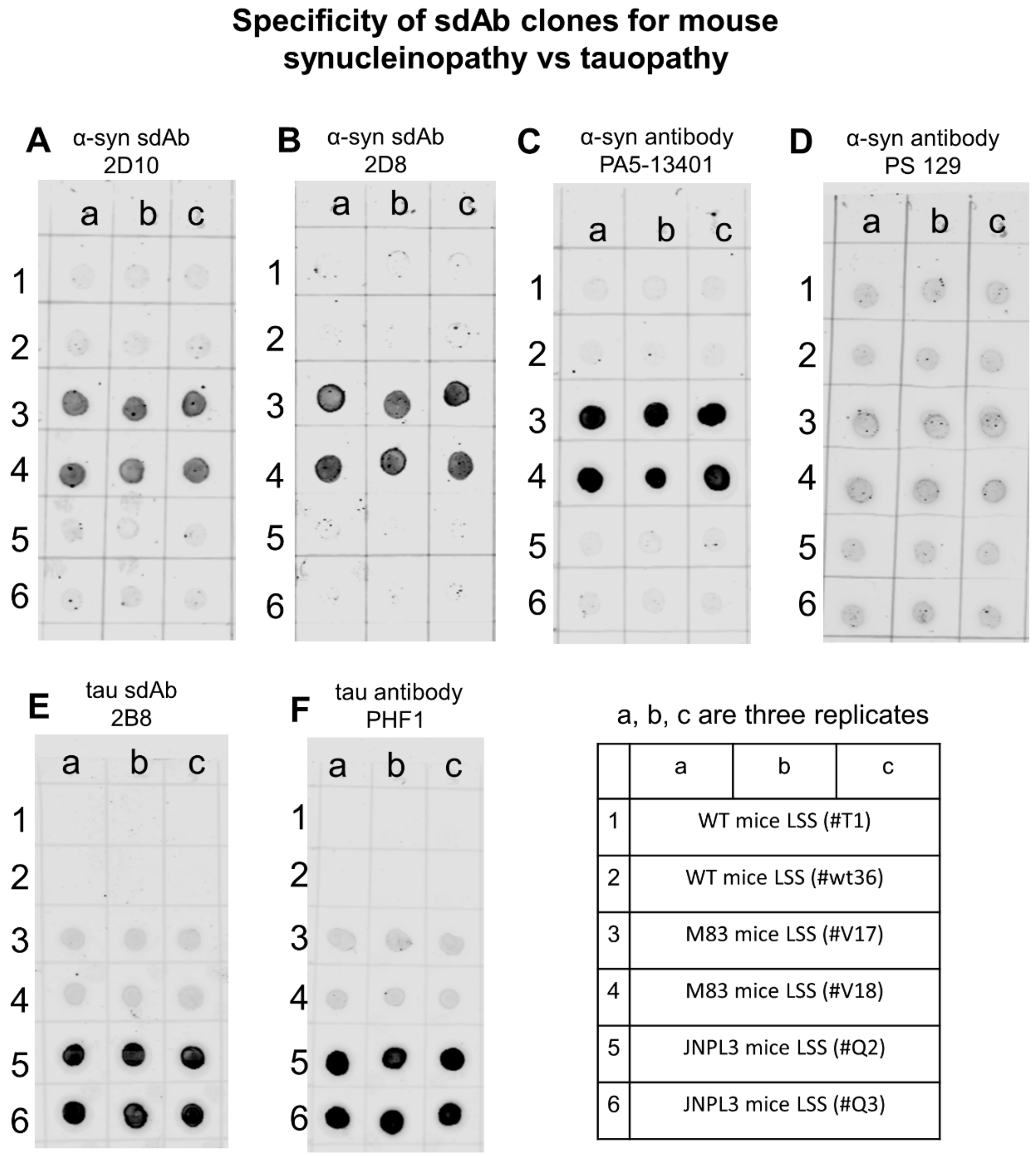

Fig. S5: Reactivity of anti-α-syn sdAbs and anti-tau sdAb to mouse synucleinopathy vs tauopathy brain tissue, compared to wild-type control brain tissue.
The sdAb antibodies and positive control antibodies were reacted with brain homogenate supernatant from two control wild-type (WT) mouse brains (lanes 1-2), two M83 synucleinopathy brains (lanes 3-4), and two JNPL3 tauopathy brains (lanes 5-6). Each sample was blotted in triplicates (a-c). The anti-α-syn sdAbs 2D10 and 2D8 are specific for synucleinopathy (A-B) and the anti-tau sdAb 2B8 is specific for tauopathy (E). The anti-α-syn sdAbs had similar reactivity as positive control anti-αsyn antibody PA5-13401 (compare A-C). The p-S129 antibody had limited reactivity in all the samples (D). The positive control tau antibody, PHF1, has similar reactivity profile as the sdAb 2B8 (compare E and F), and the M83 mice may have some tau pathology (lanes 3-4 in E and F).

sdAb affinity for α-syn targets in solid phase

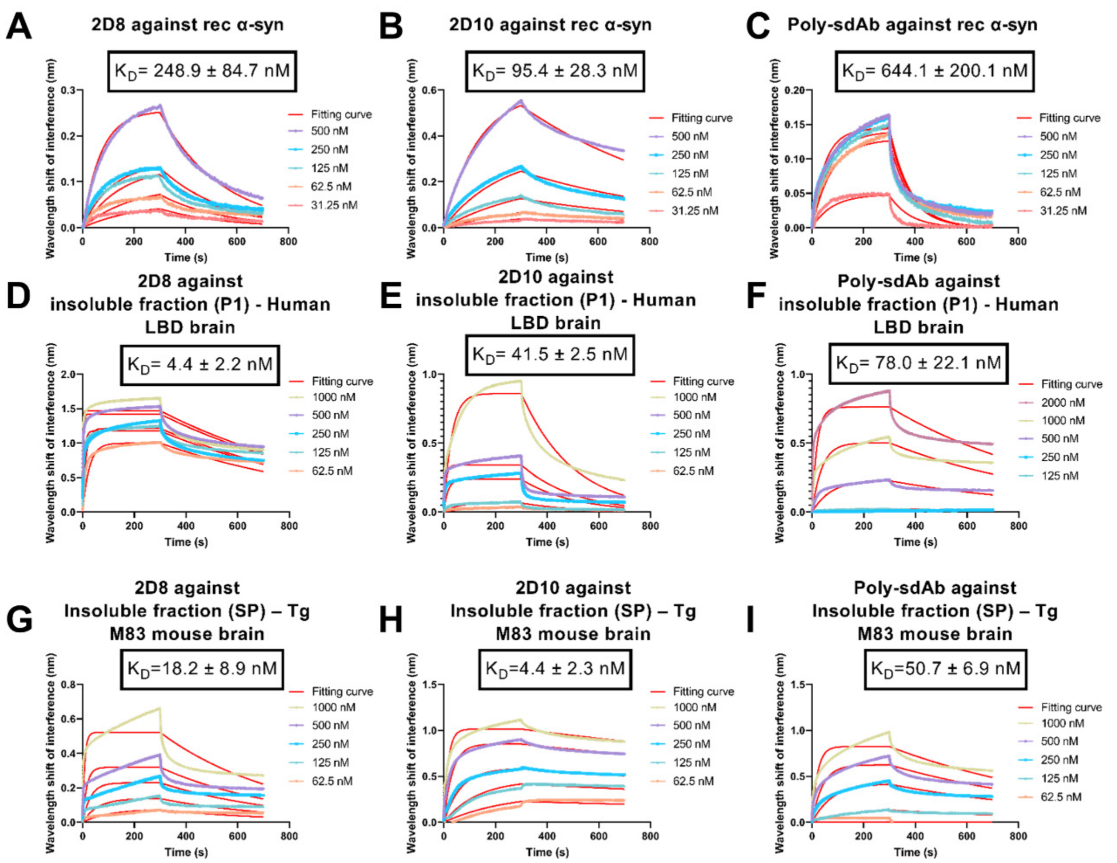

Fig. S6: Affinities of anti-α-syn sdAb 2D8, 2D10, and Poly-sdAb against different α-syn antigens (recombinant α-syn, insoluble fraction from Lewy body dementia (LBD) brain, and insoluble fraction from tg M83 mouse brain) in solid phase measured by biolayer interferometry assays.

Sensors were loaded with biotinylated forms of the different α-syn antigen preparations. K_D values were determined by measuring association and dissociation with increasing concentration of sdAb. The representative curves show the wavelength shift of interference in nanometers (nm), which is interpreted as binding. The curves show the association and dissociation of sdAb and the α-syn proteins at the different concentrations, with the red line depicting the fitting curve used to calculate the K_D value ± standard deviation (SD), which was determined from three independent experiments. See **Table S7** for the association (ka) and dissociation (kd) values.

sdAb affinity for α-syn targets in solution phase

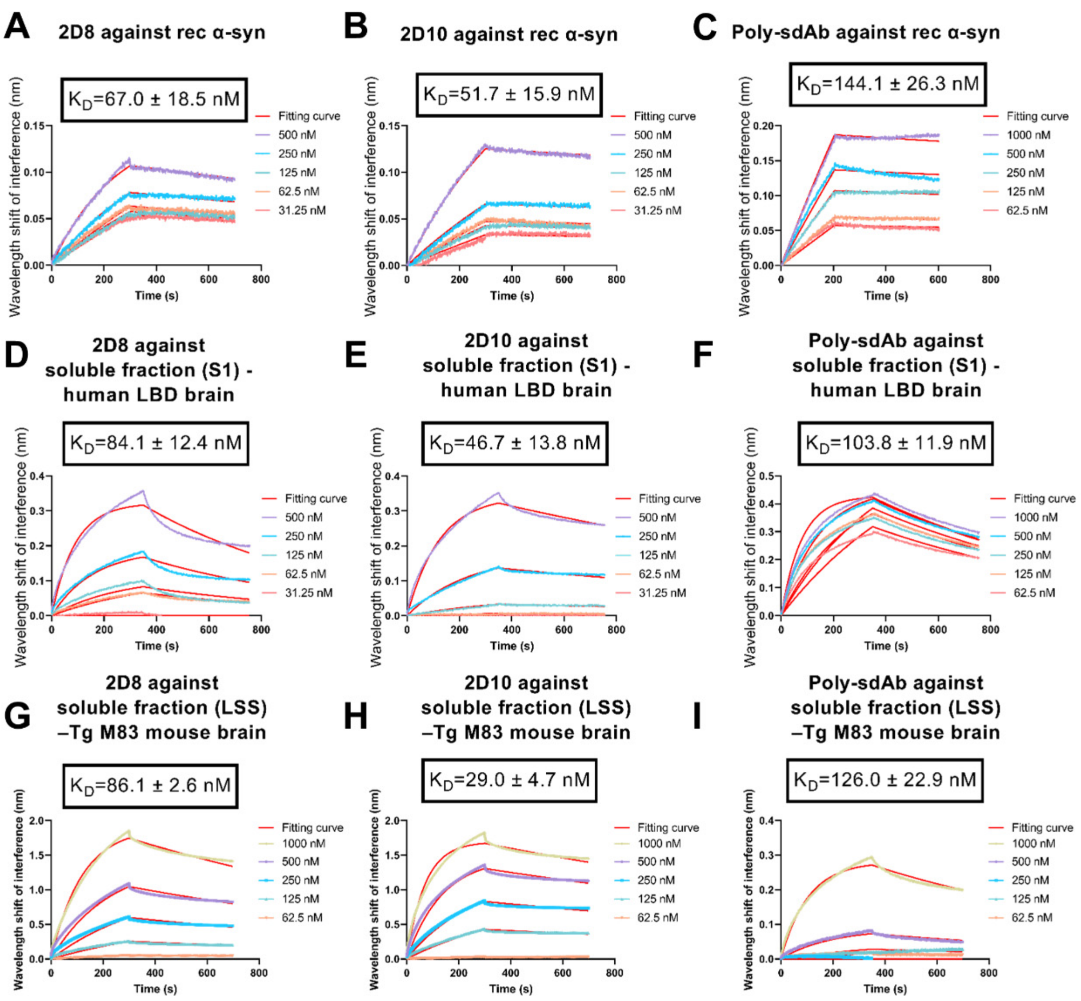

Fig. S7: Affinities of anti-α-syn sdAb 2D8, 2D10, and Poly-sdAb against different α-syn antigens (recombinant α-syn, soluble fraction from LBD brain, and soluble fraction from tg M83 mouse brain) in solution phase measured by biolayer interferometry assays. SdAbs were loaded onto the Ni-NTA biosensor. K_D values were determined using increasing concentration of the different α-syn preparations. The representative curves show the wavelength shift of interference in nanometers (nm), which is interpreted as binding. The curves show the association and dissociation of sdAb and the α-syn proteins at the different concentrations, with the red line depicting the fitting curve used to calculate the K_D value ± standard deviation (SD), which was determined from three independent experiments. See **Table S7** for the association (ka) and dissociation (kd) values.

2B8 affinity for tau targets in solid and solution phase

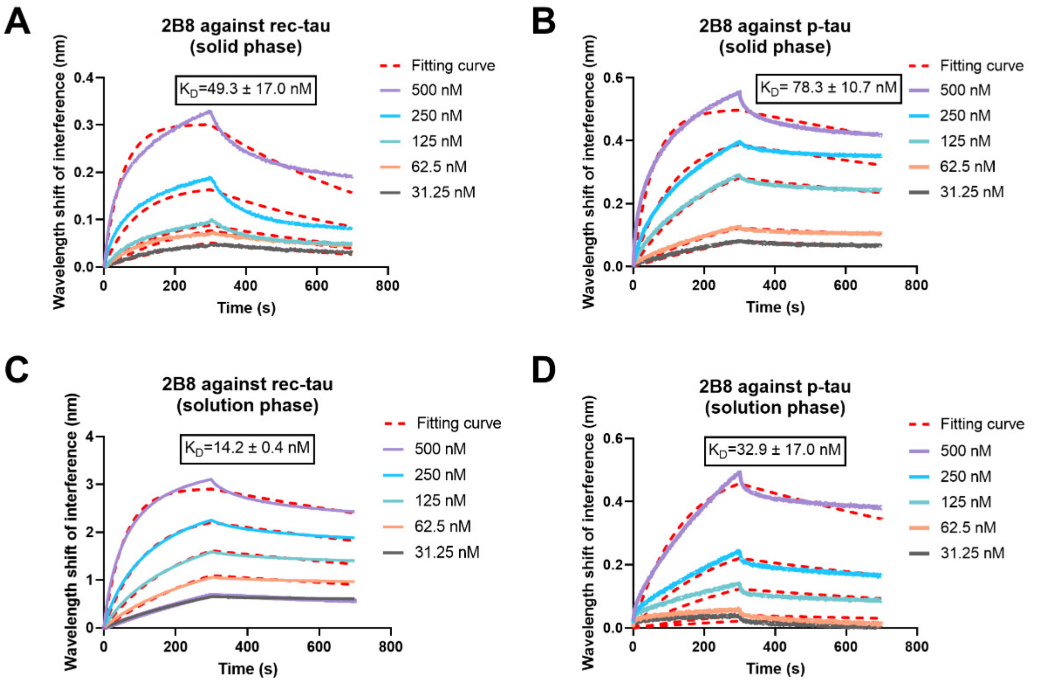

Fig. S8: Affinities of anti-tau sdAb 2B8 to recombinant longest isoform (2N4R) of human tau (rec tau) and hyperphosphorylated tau (p-tau) in solid- (A-B) and solution (C-D) phases measured by biolayer interferometry assays.

In solid phase, sensors were loaded with biotinylated forms of the different tau preparations, which are reacted with different concentrations of different sdAb. K_D values were determined using increasing concentration of the different sdAb. In the solution phase, sdAb 2B8 were loaded onto the Ni-NTA biosensor, which are reacted with different concentrations of different tau preparations. K_D values were determined using increasing concentration of the different tau preparations. The representative curves show the wavelength shift of interference in nanometers (nm), which is interpreted as binding. The curves show the association and dissociation of 2B8 and the tau proteins at the different concentrations, with the broken line depicting the fitting curve used to calculate the K_D value ± standard deviation (SD), which was determined from three independent experiments. See **Table S8** for the association (k_a) and dissociation (k_d) values.

Epitope mapping of sdAb 2D10 and 2D8 by a dot blot assay

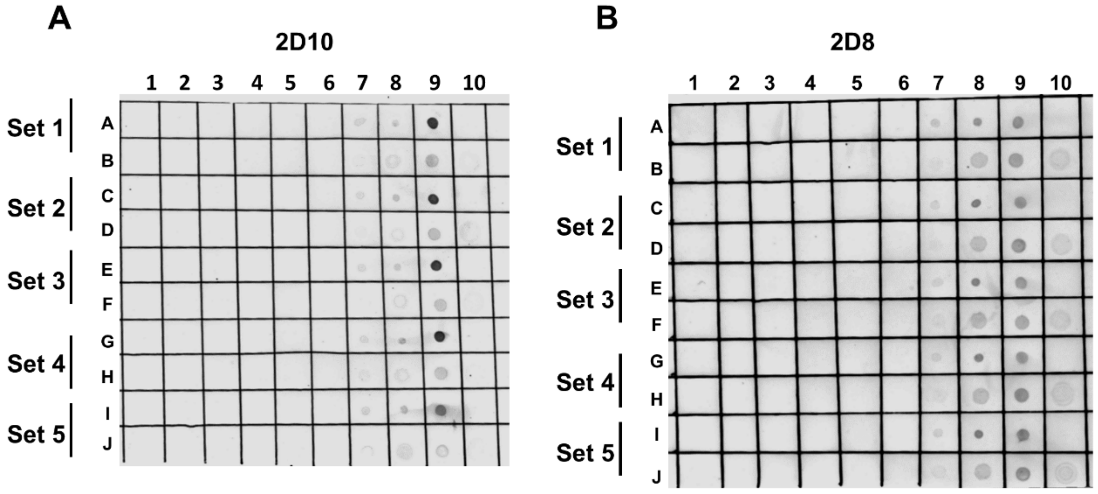

AB, CD, EF, GH, IJ, represent 5 different sets. **AB, CD, EF, GH, IJ, represent 5 different sets.**

A1	A2	A3	A4	A5	A6	A7	A8	A9	A10
Peptide 1	Peptide 2	Peptide 3	Peptide 4	Peptide 5	Peptide 6	Peptide 7	Peptide 8	Peptide 9	Peptide 10
B1	B2	B3	B4	B5	B6	B7	B8	B9	B10
Peptide 11	Peptide 12	Peptide 13	Peptide 14	Blank	BSA	S1 (LBD)	rec α-syn	SP (M83)	LSS (M83)

Fig. S9: Epitope mapping of sdAb 2D10 (A) and 2D8 (B) by a dot blot assay.
Negative control: bovine serum albumin (BSA). Positive control: α-syn preparations (human LBD brain soluble fraction S1 (LBD), rec α-syn, Tg M83 mouse human α-syn sarkosyl pellet (SP) and low speed supernatant (LSS) fractions). For peptide sequences see **Table S3**. See **Fig. 3** for quantitation and interpretation. The table clarifies the layouts of the blots in A and B, respectively.

Epitope mapping of sdAb 2B8 by a dot blot assay

A

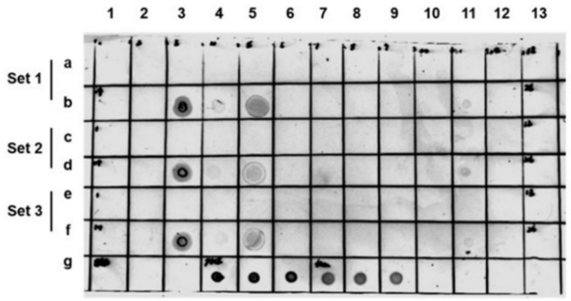

B

sdAb	2B8			
Number	A1 , C1 , E1	A2 , C2 , E2	A3 , C3 , E3	A4 , C4 , E4
Antigen	Peptide 1	Peptide 2	Peptide 3	Peptide 4
Number	A5 , C5 , E5	A6 , C6 , E6	A7 , C7 , E7	A8 , C8 , E8
Antigen	Peptide 5	Peptide 6	Peptide 7	Peptide 8
Number	A9 , C9 , E9	A10 , C10 , E10	A11 , C11 , E11	A12 , C12 , E12
Antigen	Peptide 9	Peptide 10	Peptide 11	Peptide 12
Number	A13 , C13 , E13	B1 , D1 , F1	B2 , D2 , F2	B3 , D3 , F3
Antigen	Peptide 13	Peptide 14	Peptide 15	Peptide 16
Number	B4 , D4 , F4	B5 , D5 , F5	B6 , D6 , F6	B7 , D7 , F7
Antigen	Peptide 17	Peptide 18	Peptide 19	Peptide 20
Number	B8 , D8 , F8	B9 , D9 , F9	B10 , D10 , F10	B11 , D11 , F11
Antigen	Peptide 21	Peptide 22	Peptide 23	Peptide 24
Number	B12 , D12 , F12	G1-3	G4-6	G7-9
Antigen	Peptide 25	BSA	PHF	p-tau

C

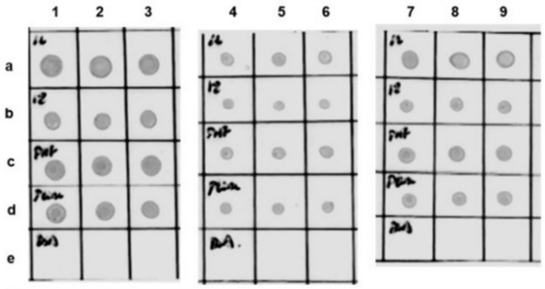

D

sdAb	2B8				
Number	a 1-3	b 1-3	c 1-3	d 1-3	e 1-3
Antigen	Peptide 16	Peptide 18	PHF	p-tau	BSA
sdAb	2B8 + peptide 16				
Antigen	a 4-6	b 4-6	c 4-6	d 4-6	e 4-6
Antigen	Peptide 16	Peptide 18	PHF	p-tau	BSA
Number	2B8 + peptide 18				
Antigen	a 7-9	b 7-9	c 7-9	d 7-9	e 7-9
Antigen	Peptide 16	Peptide 18	PHF	p-tau	BSA

Fig. S10: Epitope mapping of sdAb 2B8 by a dot blot assay.
(A-B) Epitope mapping of sdAb 2B8 using direct dot blot assay in triplicate. **(C-D)** Masking of epitope binding by pre-incubating 2B8 with ten-fold molar excess of the two peptides that reacted with 2B8. Negative control: bovine serum albumin (BSA). Positive control: paired helical filament (PHF)-enriched tau protein from human tauopathy brain and hyperphosphorylated tau (p-tau). For peptide sequences see **Table S4**. See **Fig. 11A** for quantitation and interpretation. The tables in B and D clarify the layouts of the blots in A and C, respectively.

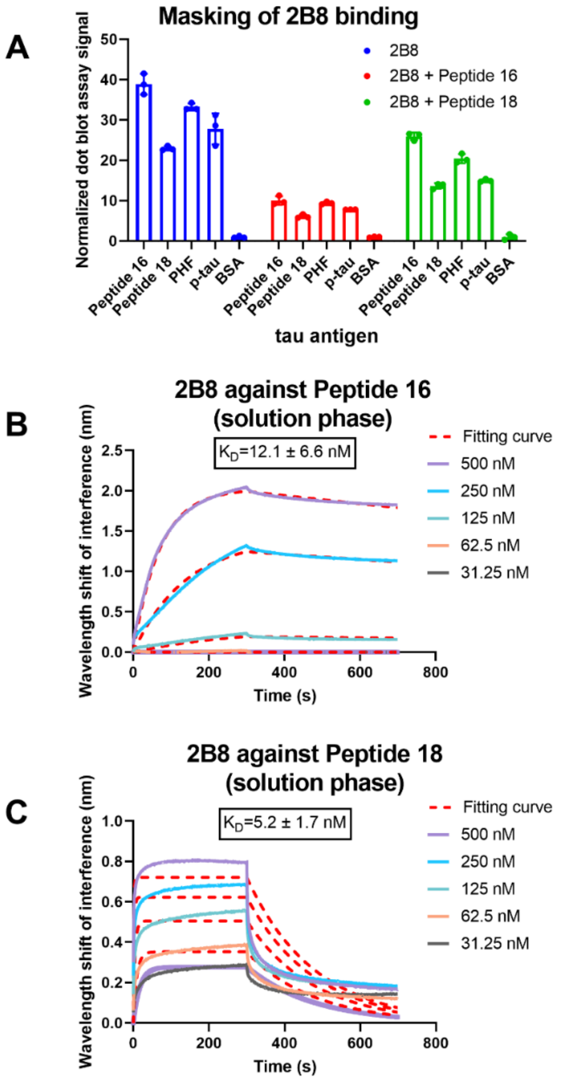

Fig. S11: Epitope mapping of sdAb 2B8.
(A) Quantification of a competitive dot blot assay of 2B8 binding. Pre-incubation of 2B8 with peptide 16 or 18 weakens the binding of 2B8 to the different tau preparations. Negative control: bovine serum albumin (BSA). Positive control: paired helical filament (PHF)-enriched tau protein from human tauopathy brain and hyperphosphorylated tau (p-tau). Each bar shows the mean normalized signal ± SD of three replicates. See **Fig. S10C** for the dot blot assay images and **Table S4** for all of the peptide sequences. (**B-C**) Binding affinities of sdAb 2B8 against epitope peptides 16 (**B**) and 18 (**C**) in the solution phase. The sdAb is bound to the biosensor, which is reacted with different concentrations of peptide 16 (**B**) and peptide 18 (**C**). The representative curves show the wavelength shift of interference in nanometers (nm), which is interpreted as binding. The curves show the association and dissociation of 2B8 and the tau peptides at the different concentrations of tau peptides, with the broken line depicting the fitting curve used to calculate the K_D value ± standard deviation (SD), which was determined from three independent experiments. See **Table S8** for the association (k_a) and dissociation (k_d) values.

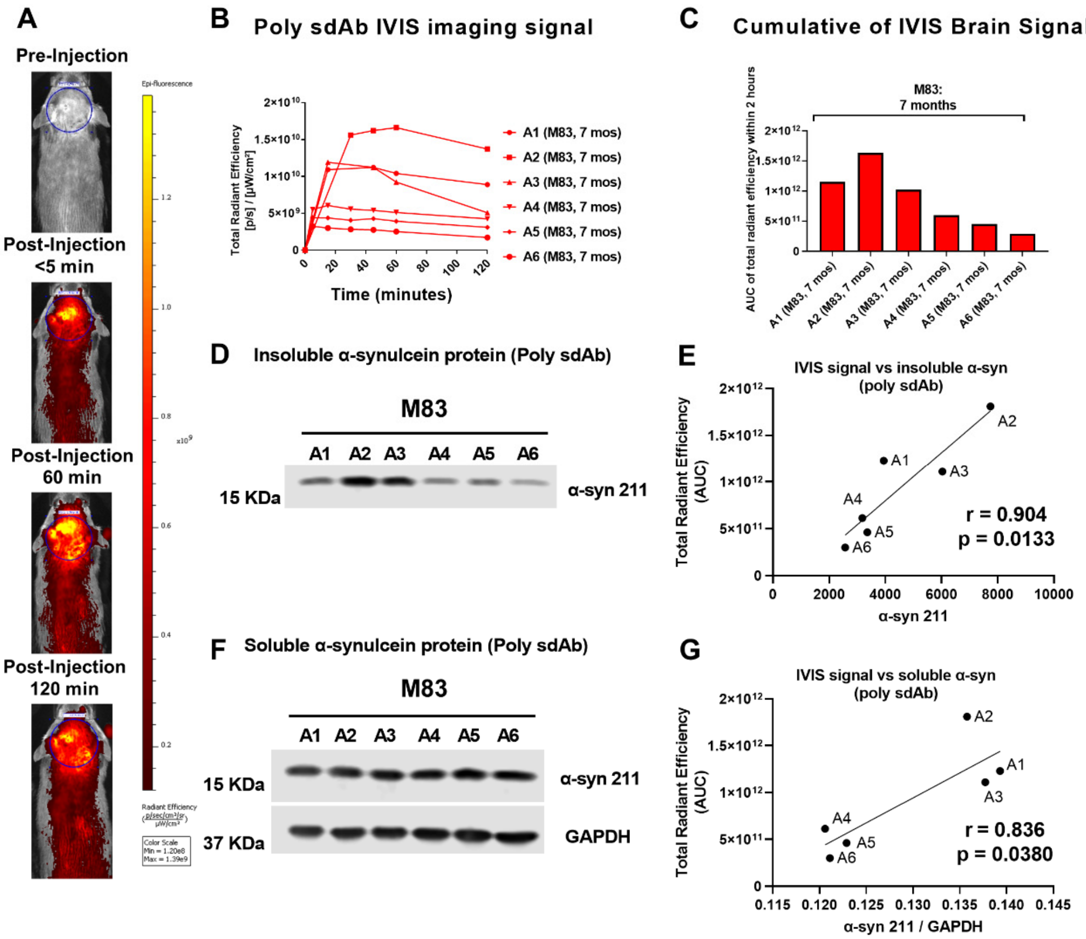

Fig. S12: Poly-sdAb in vivo imaging in M83 synucleinopathy mice.
(A) Representative mouse images of IVIS brain signal following injection with poly-sdAb tagged with a near-infrared dye (VivoTag 680 XL, 10 mg/kg). The images were recorded pre-injection and after injection at <5, 60, and 120 min. α-syn brain signal increased up to about 60 min post-injection (yellow in region of interest (ROI, circle)). Signal was detected throughout the body with highest intensity in the brain. **(B-C)** Quantitative analysis of IVIS brain signal over time (**B**) and cumulatively within 2 h post-injection (**C**). The area under the curve (AUC) is total radiant efficiency of summed pixel intensity. α-Syn brain signal was detectable right after the intravenous injection and was stable or increased up to 1-2 h post-injection. Of the sdAb signal, 24-52% remained 24 h later and was mostly cleared by 96 h (see **Fig. S13A**). **(D)** A western blot depicting insoluble α-syn protein in the brain (sarkosyl pellet reacted with α-syn 211) in M83 mice (A1-A6). **(E)** Correlation between overall IVIS brain signal with insoluble brain α-syn (r=0.904, p=0.0133). **(F)** A western blot depicting soluble α-syn protein in the same animals as in D. For correlation analysis (G), these levels were normalized to GAPDH levels. **(G)** Correlation between overall IVIS brain signal with soluble brain α-syn (r=0.836, p=0.0380).

Brain imaging signal over several days after intravenous injection of sdAbs.

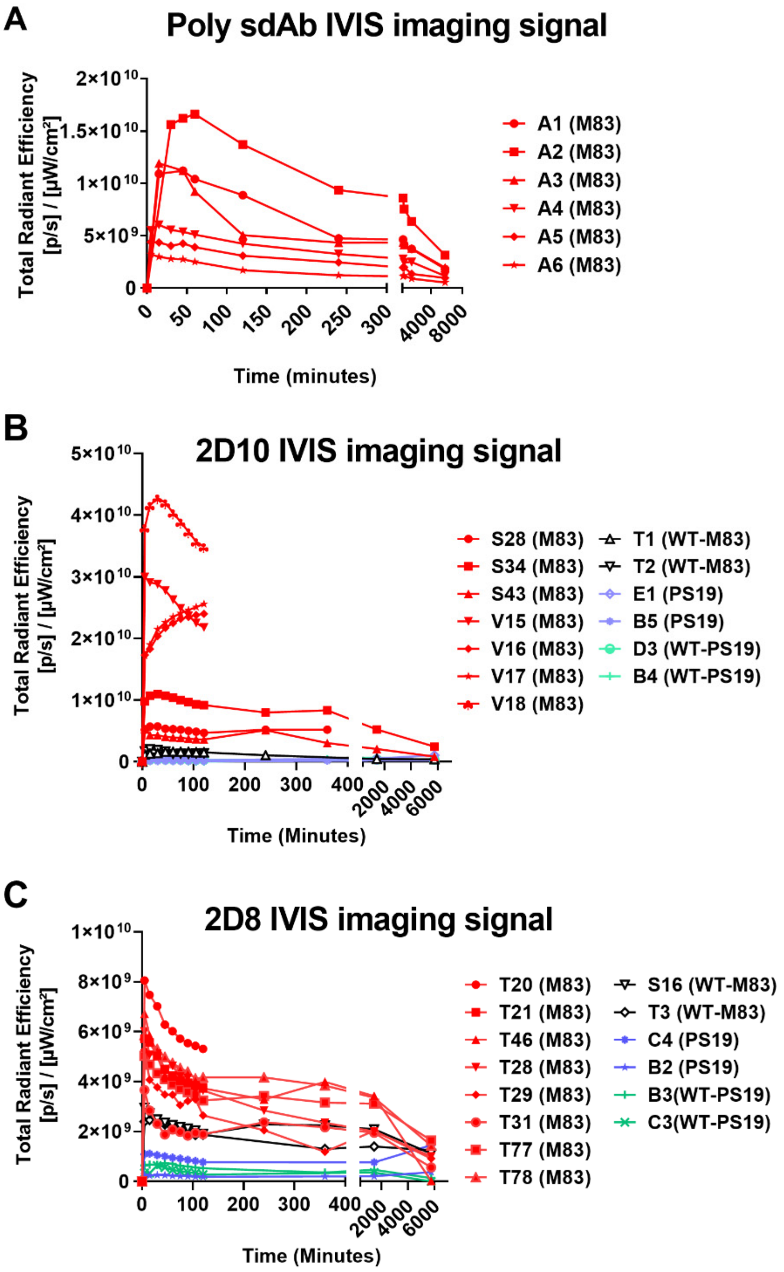

Fig. S13: Brain imaging signal over several days after intravenous injection of sdAbs. **(A)** IVIS poly sdAb brain signal typically peaked in M83 α-syn mice within 1 h after intravenous injection (10 mg/kg). **(B)** IVIS 2D10 sdAb brain signal was specific for M83 α-syn mice, and typically peaked within 2 h after intravenous injection (10 mg/kg). **(C)** IVIS 2D8 sdAb signal in the brain was specific for M83 α-syn mice, and typically peaked within 2 h after intravenous injection (10 mg/kg). The signal of all three probes gradually decreased after 1-2 h but was still detectable in most α-syn animals 96 h post-injection. A few animals in B and C were killed at 2 h for histological brain analysis (see Figures 8-9).

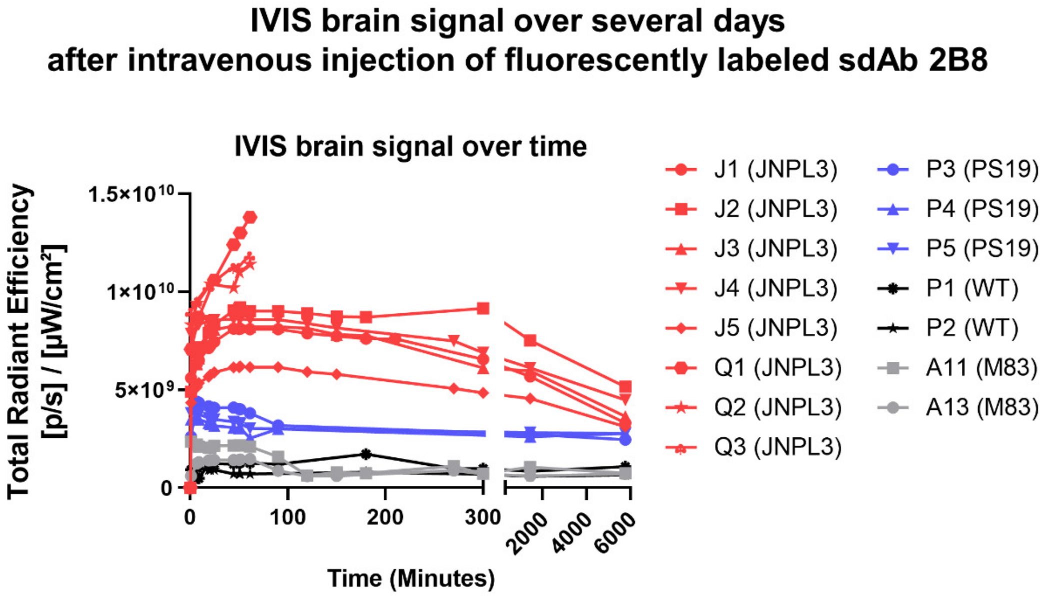

Fig. S14: IVIS brain signal over several days after intravenous injection of fluorescently labeled sdAb 2B8.

IVIS sdAb 2B8 signal in the brain is specific for tauopathy, and typically peaks at about 1 h after intravenous injection (10 mg/kg) but is still detected in the brains to a lesser degree up to 100 h after administration. A few animals were killed at 1 h for histological analysis (see Figure 10).

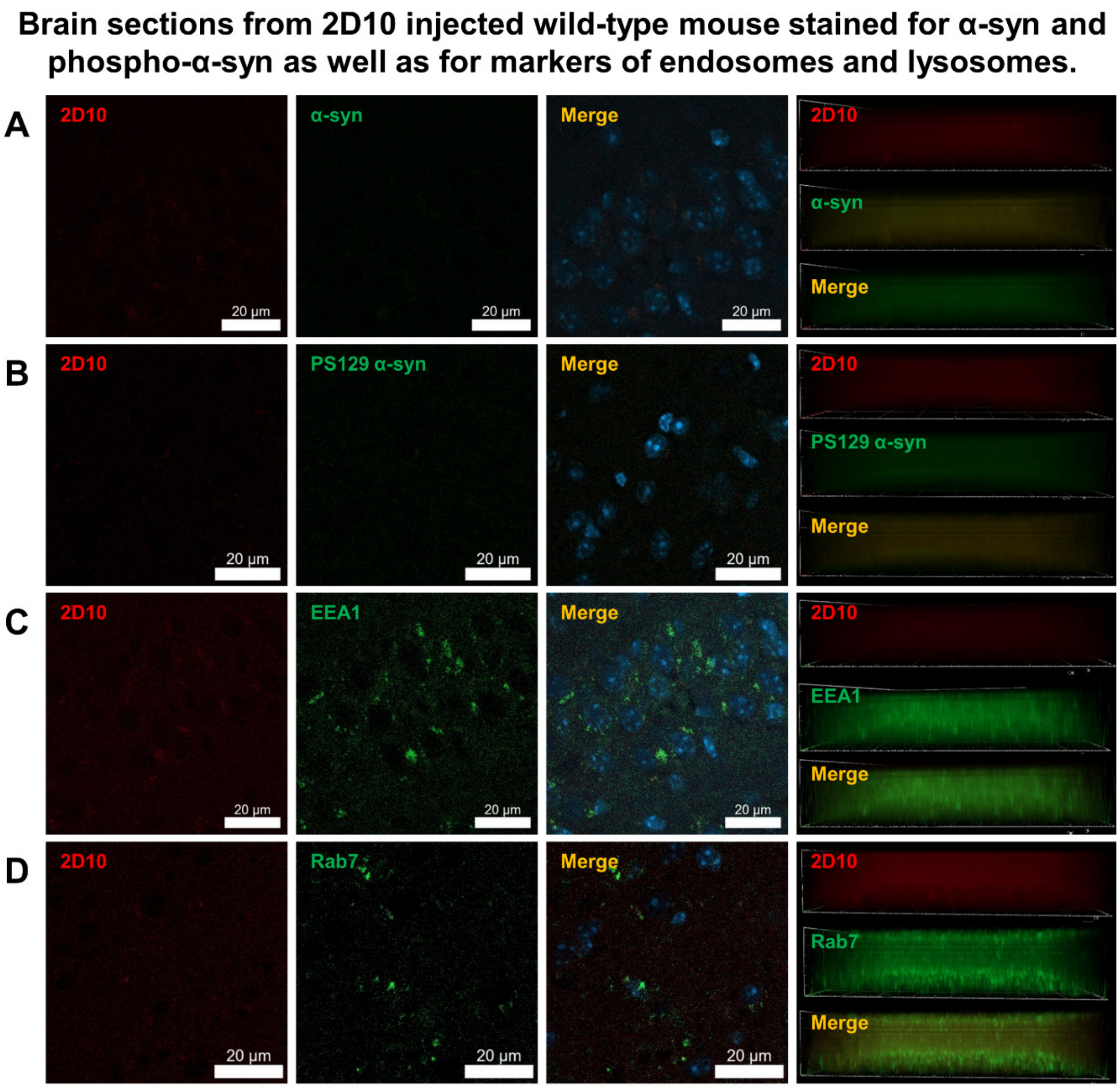

Fig. S15: Brain sections from 2D10 injected wild-type mouse stained for α-syn and phospho-α-syn as well as for markers of endosomes and lysosomes.

Near-infrared tag labeled sdAb 2D10 was injected intravenously (i.v.), the brains were perfused with PBS and removed 2 h after injection, post-fixed, sectioned and stained with a nuclear stain (Hoechst, blue), and an antibody against (A) α-syn (α-syn 211) or (B) phospho-serine 129 α-syn (PS129 α-syn), (C) early endosomes (EEA1 and (D) late endosomes/lysosomes (Rab7). Limited if any 2D10 signal was detected in the WT brains as shown in the representative coronal sections from wild-type mouse T2 in the three panels from the left and confirmed in the Z-stacks (right panel). As expected, these brains did not have α-syn aggregates but did stain with the EEA and Rab7 markers. Scale bar = 20 μm.

Brain sections from 2D8 injected wild-type mouse stained for α-syn and phospho-α-syn as well as for markers of endosomes and lysosomes.

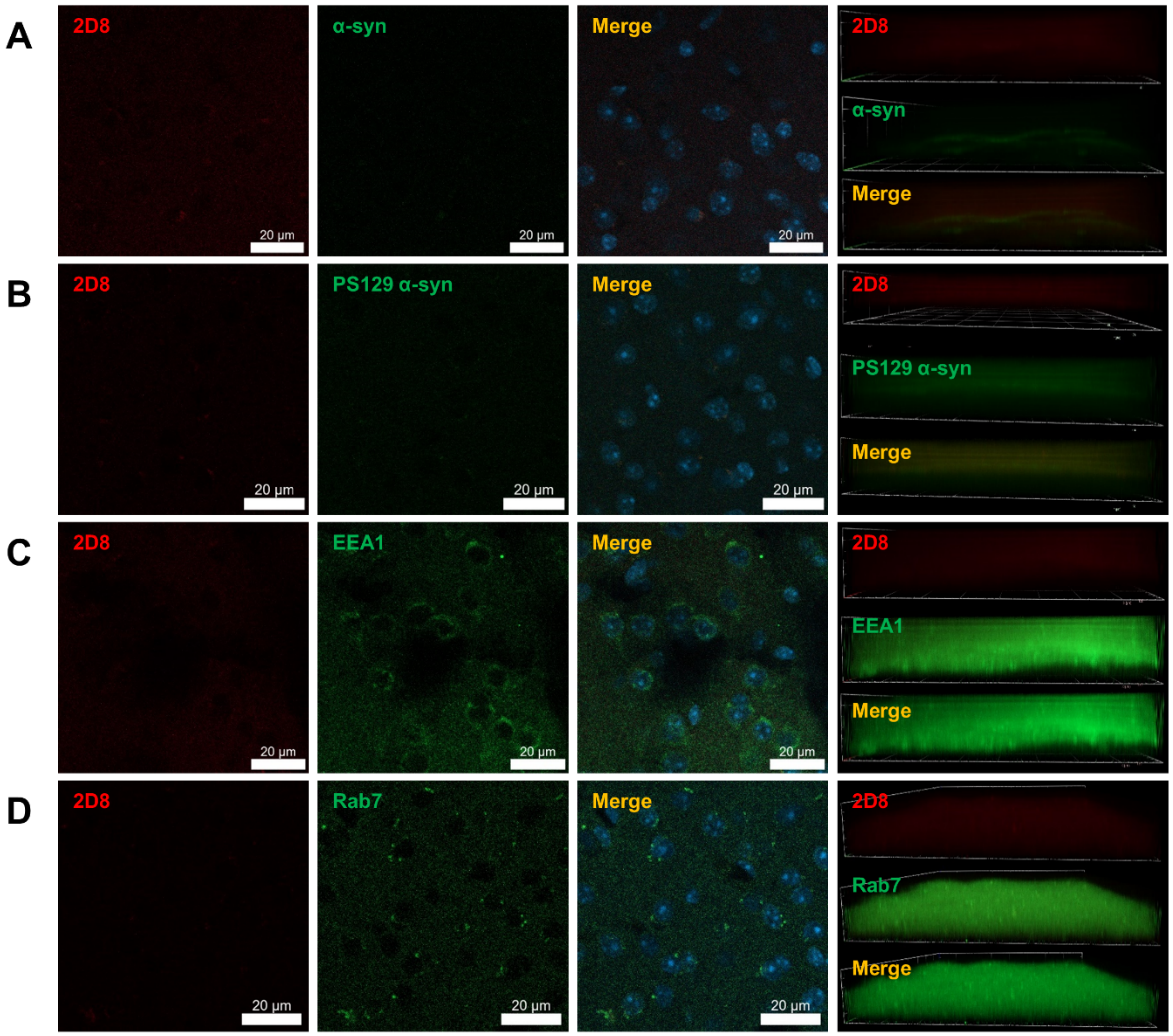

Fig. S16: Brain sections from 2D8 injected wild-type mouse stained for α-syn and phospho-α-syn as well as for markers of endosomes and lysosomes.
Near-infrared tag labeled sdAb 2D8 was injected intravenously (i.v.), the brains were perfused with PBS and removed 2 h after injection, post-fixed, sectioned and stained with a nuclear stain (Hoechst, blue), and an antibody against (A) α-syn (α-syn 211) or (B) phospho-serine 129 α-syn (PS129 α-syn), (C) early endosomes (EEA1 and (D) late endosomes/lysosomes (Rab7). Limited if any 2D8 signal was detected in the WT brains as shown in the representative coronal sections from wild-type mouse S16 in the three panels from the left and confirmed in the Z-stacks (right panel). As expected, these brains did not have α-syn aggregates but did stain with the EEA and Rab7 markers. Scale bar = 20 µm.

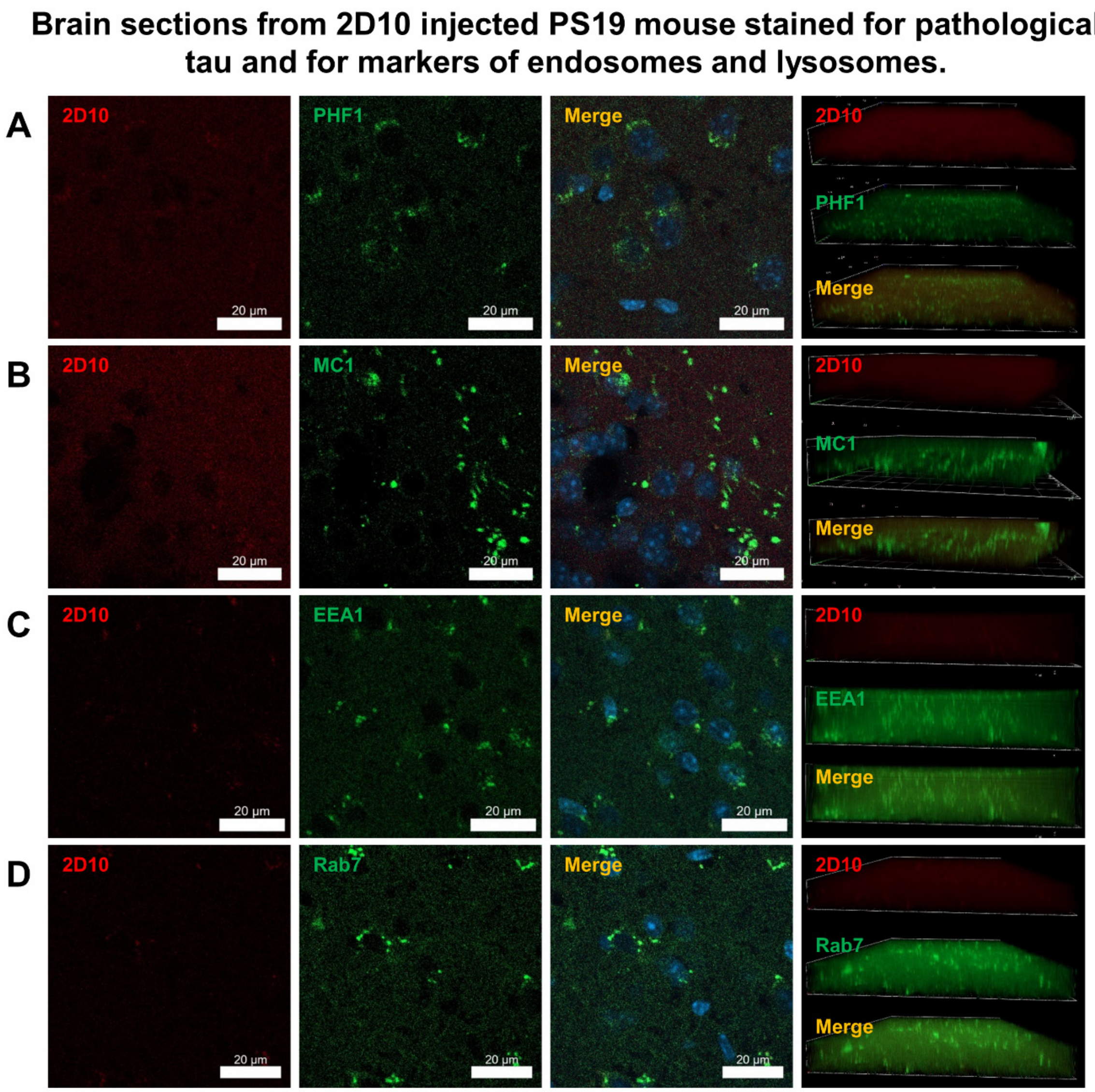

Fig. S17: Brain sections from 2D10 injected PS19 tauopathy mouse stained for anti-tau PHF1 and MC1 antibody as well as for markers of endosomes and lysosomes.
Near-infrared tag labeled sdAb 2D10 was injected intravenously (i.v.), the brains were perfused with PBS and removed 2 h after injection, post-fixed, sectioned and stained with a nuclear stain (Hoechst, blue), and an antibody against (A) hyperphosphorylated tau (PHF1) or (B) a conformational tau epitope (MC1), (C) early endosomes (EEA1 and (D) late endosomes/lysosomes (Rab7). Limited if any 2D10 signal was detected in the PS19 brains as shown in the representative coronal sections from PS19 mouse E1 in the three panels from the left and confirmed in the Z-stacks (right panel). Scale bar = 20 µm.

Brain sections from 2D8 injected PS19 mouse stained for pathological tau and for markers of endosomes and lysosomes.

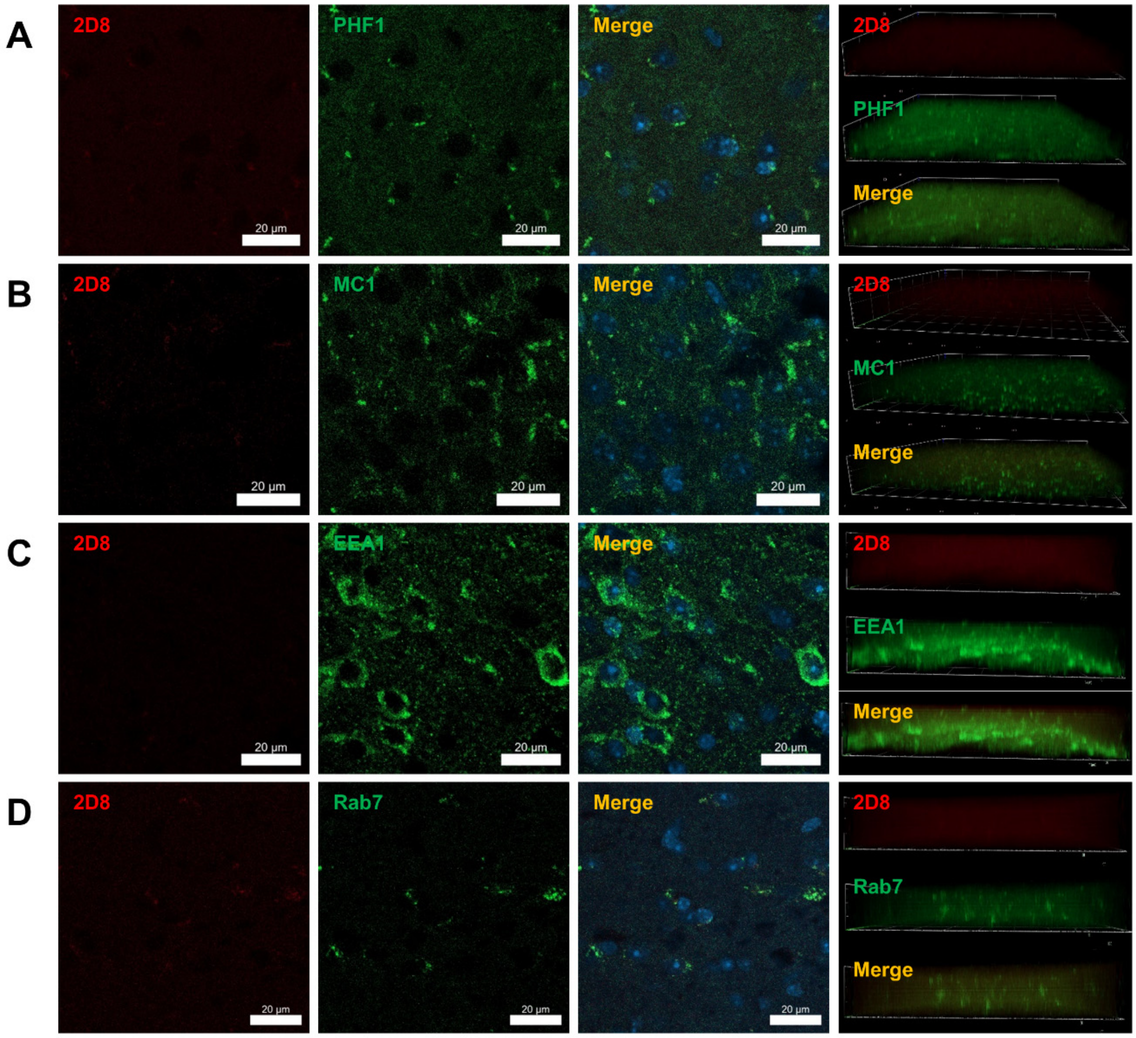

Fig. S18: Brain sections from 2D8 injected PS19 tauopathy mouse stained for anti-tau PHF1 and MC1 antibody as well as for markers of endosomes and lysosomes.
Near-infrared tag labeled sdAb 2D8 was injected intravenously (i.v.), the brains were perfused with PBS and removed 2 h after injection, post-fixed, sectioned and stained with a nuclear stain (Hoechst, blue), and an antibody against (A) hyperphosphorylated tau (PHF1) or (B) a conformational tau epitope (MC1), (C) early endosomes (EEA1 and (D) late endosomes/lysosomes (Rab7). Limited if any 2D8 signal was detected in the PS19 brains as shown in the representative coronal sections from PS19 mouse C4 in the three panels from the left and confirmed in the Z-stacks (right panel). Scale bar = 20 µm.

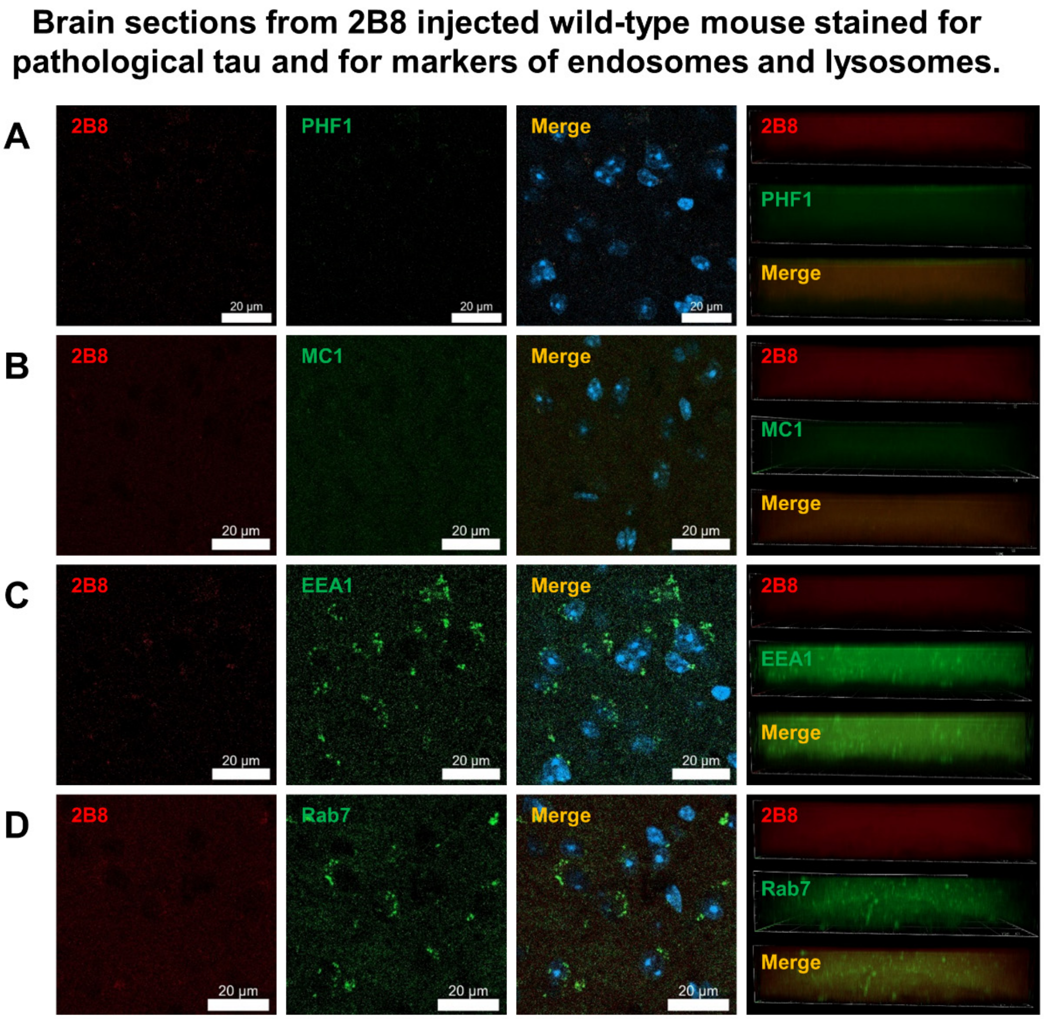

Fig. S19: Brain sections from 2B8 injected WT mouse stained with tau and endosomal-lysosomal markers to assess the presence of 2B8 within the brain and its cellular location. Near-infrared dye labeled sdAb 2B8 was injected intravenously (i.v.), the brains were perfused with PBS and removed 1 h after injection, post-fixed, sectioned and stained with a nuclear stain (Hoechst), and an antibody against (A) hyperphosphorylated tau (PHF1) or (B) a conformational tau epitope (MC1), (C) early endosomes (EEA1 and (D) late endosomes/lysosomes (Rab7). Limited if any 2B8 signal was detected in the WT brains as shown in the representative coronal sections from wt36 in the top panels and confirmed in the Z-stacks (right panel). As expected, these brains did not have pathological tau but did stain with the EEA and Rab7 markers. Scale bar = 20 µm.

Brain sections from 2B8 injected M83 mouse stained for α-syn and phospho-α-syn as well as for markers of endosomes and lysosomes

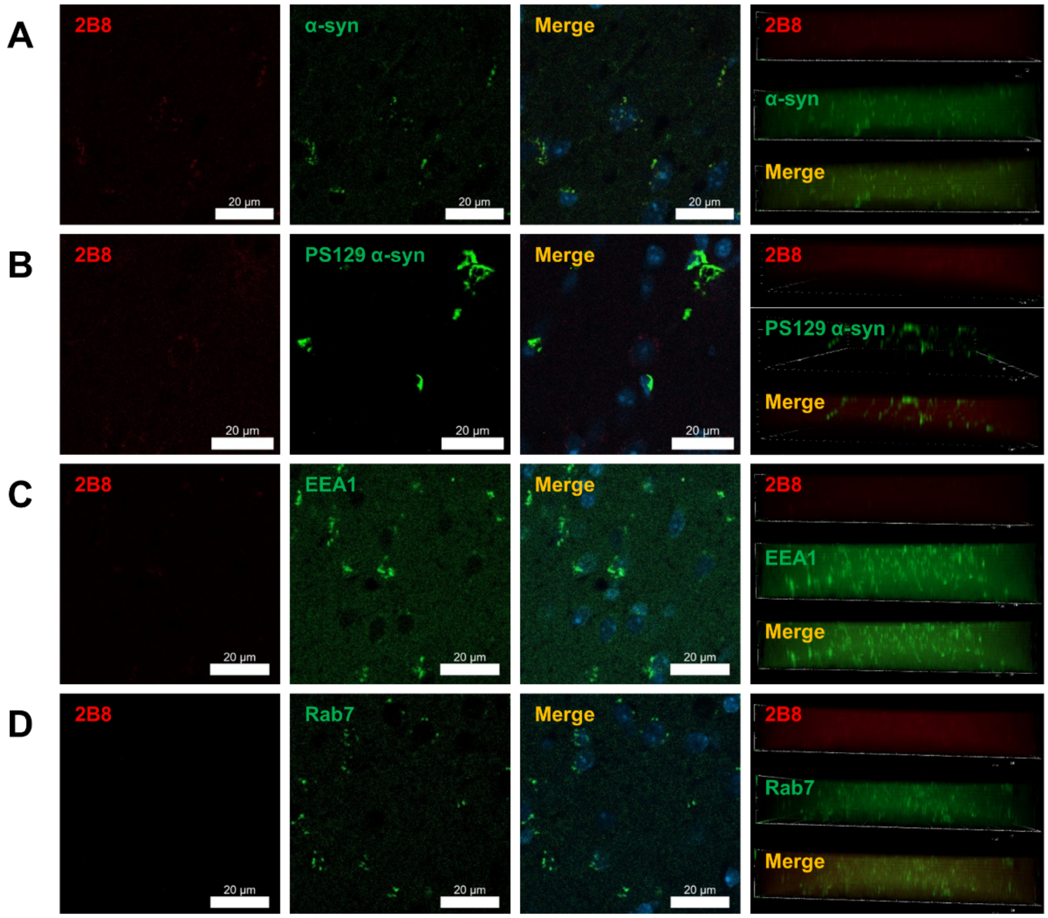

Fig. S20: Brain sections from 2B8 injected M83 mouse stained with α-syn and phospho-α-syn as well as for markers of endosomes and lysosomes.
Near-infrared dye labeled sdAb 2B8 was injected intravenously (i.v.), the brains were perfused with PBS and removed 1 h after injection, post-fixed, sectioned and stained with a nuclear stain (Hoechst), and an antibody against (A) α-syn (α-syn 211) or (B) phospho-serine 129 α-syn (PS129 α-syn), (C) early endosomes (EEA1 and (D) late endosomes/lysosomes (Rab7). Limited if any 2B8 signal was detected in the M83 mouse brains as shown in the representative coronal sections from M83 mouse V23 in the three panels from the left and confirmed in the Z-stacks (right panel). Scale bar = 20 µm.

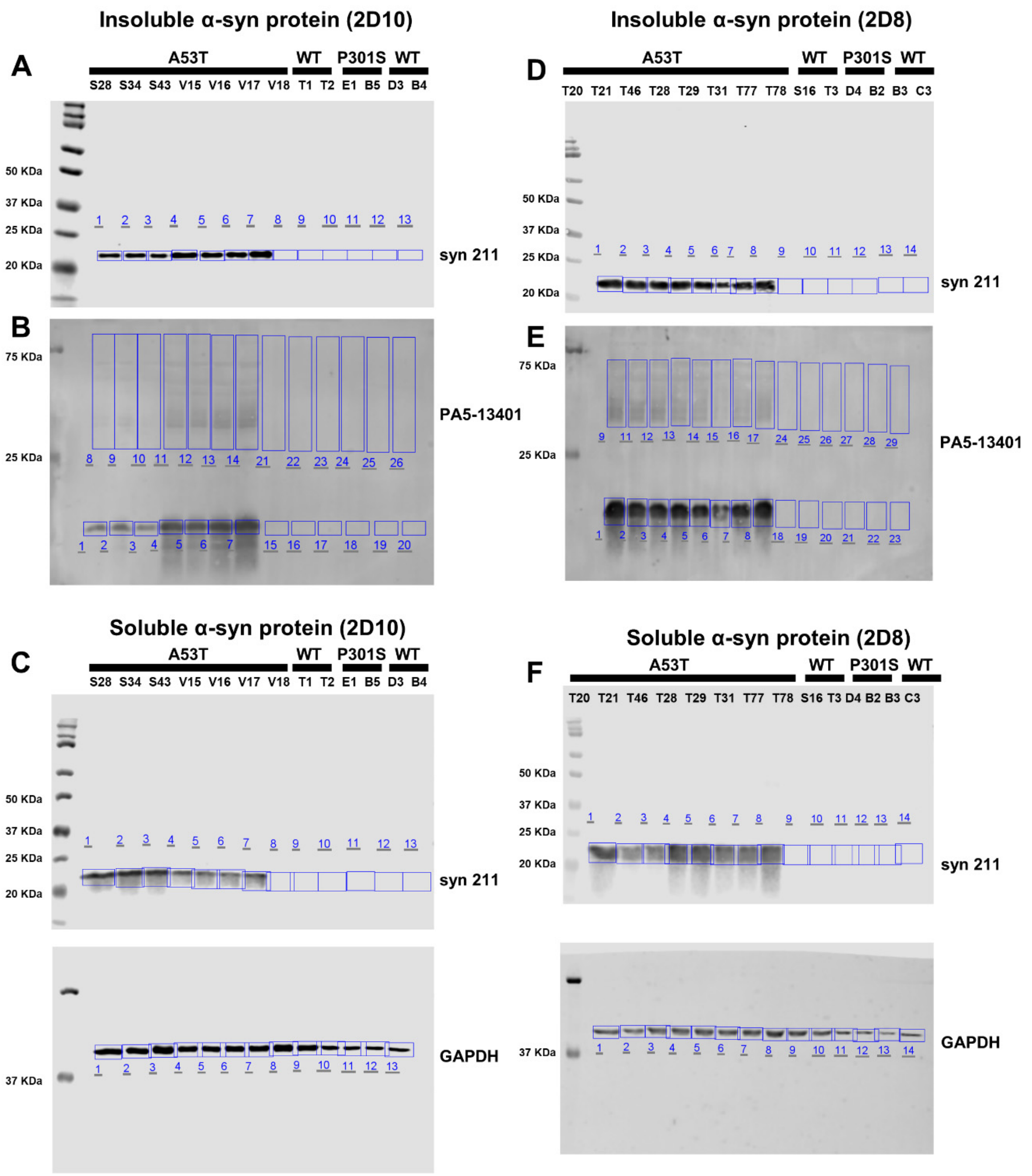

Fig. S21: Complete α-syn western blots and bands quantified in Fig. 5.

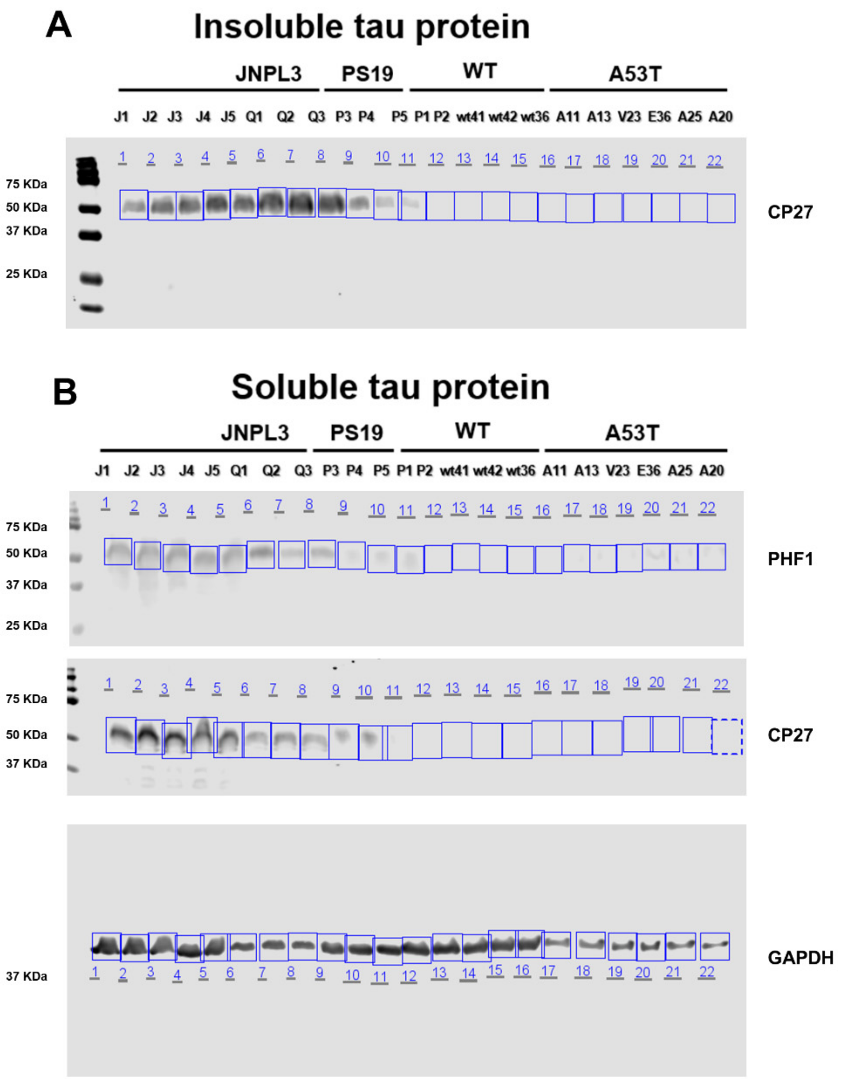

Fig. S22: Complete tau western blots and bands quantified in Fig. 7.

Procedure	Route	Vaccine	Day
Pre-bleed			0
Immunization 1	SQ	200µg α-syn in CFA	0
Immunization 2	SQ	100µg α-syn in IFA	21
Bleed 1			28
Immunization 3	SQ	100µg α-syn in IFA + 100µg α-syn in Adjuplex	49
Bleed 2			56
Immunization 4	SQ	100µg α-syn in IFA + 100µg α-syn in Adjuplex	77
Bleed 3			84
Immunization 5	SQ	100µg α-syn in IFA + 100µg α-syn in Adjuplex	105
Bleed 4 (1 x 10^8 PBMCs)			112
Immunization 6	Split SQ / IM	200µg α-syn in Adjuplex	144
Bleed 5 (1 x 10^8 PBMCs)			151
Immunization 7	Split SQ / IM	200µg α-syn in Adjuplex	188
Bleed 6			196

Table S1: Immunization protocol for the llama from which anti-α-syn sdAbs were derived. Abbreviations: Recombinant human α-synuclein (α-syn), Complete Freund's Adjuvant (CFA), Incomplete Freund's Adjuvant (IFA), Subcutaneous (SQ), Intramuscular (IM), Peripheral Blood Mononuclear Cells (PBMCs), Adjuplex Adjuvant (Sigma).

Procedure	Route	Vaccine	Day
Pre-bleed			0
Immunization 1	SQ	200µg Tau in CFA	0
Immunization 2	SQ	100µg Tau in IFA	21
Bleed 1			28
Immunization 3	SQ	100µg Tau in IFA + 100µg Tau in Adjuplex	49
Bleed 2			56
Immunization 4	SQ	100µg Tau in IFA + 100 µg Tau in Adjuplex	77
Bleed 3			84
Immunization 5	SQ	100µg Tau in IFA + 100µg Tau in Adjuplex	105
Bleed 4 (1 x 10^8 PBMCs)			112
Immunization 6	Split SQ / IM	200 µg PHF in Adjuplex	142
Bleed 5			149
Immunization 7	Split SQ / IM	200 µg PHF in Adjuplex	188
Bleed 6 (1 x 10^8 PBMCs)			196

Table S2: Immunization protocol for the llama from which anti-tau sdAbs were derived. Abbreviations: Recombinant longest isoform of human tau (Tau, 2N4R), Complete Freund's Adjuvant (CFA, Sigma), Incomplete Freund's Adjuvant (IFA, Sigma), Subcutaneous (SQ), Intramuscular (IM), Peripheral Blood Mononuclear Cells (PBMCs), PHF is paired helical filament-enriched tau protein from human tauopathy brain. Adjuplex Adjuvant (Sigma).

Peptide	Sequence
Peptide 1 (α-syn 1-15)	MDVFMKGLSKAKEGV
Peptide 2 (α-syn 11-25)	AKEGVVAAAEKTKQG
Peptide 3 (α-syn 21-35)	KTKQGVAEAAGKTKE
Peptide 4 (α-syn 31-45)	GKTKEGVLYVGSKTK
Peptide 5 (α-syn 41-55)	GSKTKEGVVHGVATV
Peptide 6 (α-syn 51-65)	GVATVAEKTKEQVTN
Peptide 7 (α-syn 61-75)	EQVTNVGGAVVTGVT
Peptide 8 (α-syn 71-85)	VTGVTAVAQKTVEGA
Peptide 9 (α-syn 81-95)	TVEGAGSIAAATGFV
Peptide 10 (α-syn 91-105)	ATGFVKKDQLGKNEE
Peptide 11 (α-syn 101-115)	GKNEEGAPQEGILED
Peptide 12 (α-syn 111-125)	GILEDMPVDPDNEAY
Peptide 13 (α-syn 121-135)	DNEAYEMPSEEGYQD
Peptide 14 (α-syn 131-140)	EGYQDYEPEA

Table S3: α-Synuclein peptide library for epitope mapping.
To mimic the charge state in the native protein, peptides 2-14 are acetylated on the N-terminus and peptides 1-13 are amidated on their C-terminus.

Peptide No.	Sequence
Peptide 1 (tau 1-25)	MAEPRQEFEVMEDHAGTYGLGDRKD
Peptide 2 (tau 19-43)	GLGDRKDQGGYTMHQDQEGDTDAGL
Peptide 3 (tau 37-61)	GDTDAGLKESPLQTPTEDGSEEPGS
Peptide 4 (tau 55-79)	GSEEPGSETSDAKSTPTAEDVTAPL
Peptide 5 (tau 73-97)	EDVTAPLVDEGAPGKQAAAQPHTEI
Peptide 6 (tau 91-115)	AQPHTEIPEGTTAEEAGIGDTPSLE
Peptide 7 (tau 109-133)	GDTPSLEDEAAGHVTQARMVSKSKD
Peptide 8 (tau 127-157)	MVSKSKDGTGSDDKKAKGADGKTKI
Peptide 9 (tau 145-169)	ADGKTKIATPRGAAPPGQKGQANAT
Peptide 10 (tau 163-187)	KGQANATRIPAKTPPAPKTPPSSGE
Peptide 11 (tau 181-205)	TPPSSGEPPKSGDRSGYSSPGSPGT
Peptide 12 (tau 199-203)	SPGSPGTPGSRSRTPSLPTPPTREP
Peptide 13 (tau 217-241)	TPPTREPKKVAVVRTPPKSPSSAKS
Peptide 14 (tau 235-259)	SPSSAKSRLQTAPVPMPDLKNVKSK
Peptide 15 (tau 253-277)	LKNVKSKIGSTENLKHQPGGGKVQI
Peptide 16 (tau 271-295)	GGGKVQIINKKLDLSNVQSKCGSKD
Peptide 17 (tau 289-313)	SKCGSKDNIKHVPGGGSVQIVYKPV
Peptide 18 (tau 307-331)	QIVYKPVDLSKVTSKCGSLGNIHHK
Peptide 19 (tau 325-349)	LGNIHHKPGGGQVEVKSEKLDFKDR
Peptide 20 (tau 343-367)	KLDFKDRVQSKIGSLDNITHVPGGG
Peptide 21 (tau 361-385)	THVPGGGNKKIETHKLTFRENAKAK
Peptide 22 (tau 379-403)	RENAKAKTDHGAEIVYKSPVVSGDT
Peptide 23 (tau 397-421)	PVVSGDTSPRHLSNVSSTGSIDMVD
Peptide 24 (tau 415-439)	GSIDMVDSPQLATLADEVSASLAKQ
Peptide 25 (tau 433-441)	SASLAKQGL

Table S4: Tau peptide library for epitope mapping.
To mimic the charge state in the native protein, peptides 2-25 are acetylated on the N-terminus and peptides 1-24 are amidated on their C-terminus. Sequence homology between peptides 16 and 18 is shown in red.

sdAb	Mouse #	Model	Age (months)	Gender
2D8	T20	M83 (α-syn A53T)	8	F
2D8	T21	M83 (α-syn A53T)	8	F
2D8	T46	M83 (α-syn A53T)	8	F
2D8	T28	M83 (α-syn A53T)	8	F
2D8	T29	M83 (α-syn A53T)	8	F
2D8	T31	M83 (α-syn A53T)	8	F
2D8	T77	M83 (α-syn A53T)	6	M
2D8	T78	M83 (α-syn A53T)	7	M
2D8	S16	WT (M83 strain)	6	M
2D8	T3	WT (M83 strain)	8	F
2D8	D4	PS19 (tau P301S)	10	M
2D8	B2	PS19 (tau P301S)	7	F
2D8	B3	WT (PS19 strain)	7	F
2D8	C3	WT (PS19 strain)	10	M
**sdAb**	**Mouse #**	**Model**	**Age (months)**	**Gender**
2D10	S28	M83 (α-syn A53T)	6	M
2D10	S34	M83 (α-syn A53T)	6	M
2D10	S43	M83 (α-syn A53T)	8	F
2D10	V15	M83 (α-syn A53T)	5	F
2D10	V16	M83 (α-syn A53T)	7	M
2D10	V17	M83 (α-syn A53T)	7	M
2D10	V18	M83 (α-syn A53T)	6	M
2D10	T1	WT (M83 strain)	6	M
2D10	T2	WT (M83 strain)	7	F
2D10	E1	PS19 (tau P301S)	10	M
2D10	B5	PS19 (tau P301S)	9	F
2D10	D3	WT (PS19 strain)	10	M
2D10	B4	WT (PS19 strain)	10	F
**sdAb**	**Mouse #**	**Model**	**Age (months)**	**Gender**
Poly	A1	M83 (α-syn A53T)	7	F
Poly	A2	M83 (α-syn A53T)	7	F
Poly	A3	M83 (α-syn A53T)	7	F
Poly	A4	M83 (α-syn A53T)	7	F
Poly	A5	M83 (α-syn A53T)	7	F
Poly	A6	M83 (α-syn A53T)	7	F

Table S5: List of the mice injected with labeled sdAbs 2D8, 2D10 or the polyclonal sdAb that these two monoclonals were derived from (10 mg/kg each).
The wild-type (WT) mice were of the same strain background as the M83 (α-syn A53T) and the PS19 (tau P301S) models.

sdAb	Mouse #	Model	Age (months)	Gender
2B8	J1	JNPL3 (tau P301L)	17	M
2B8	J2	JNPL3 (tau P301L)	17	M
2B8	J3	JNPL3 (tau P301L)	17	M
2B8	J4	JNPL3 (tau P301L)	17	M
2B8	J5	JNPL3 (tau P301L)	17	M
2B8	Q1	JNPL3 (tau P301L)	17	M
2B8	Q2	JNPL3 (tau P301L)	17	M
2B8	Q3	JNPL3 (tau P301L)	17	M
2B8	wt41	WT (JNPL3 strain)	15	F
2B8	wt42	WT (JNPL3 strain)	15	F
2B8	wt36	WT (JNPL3 strain)	18	F
2B8	P3	PS19 (tau P301S)	9	M
2B8	P4	PS19 (tau P301S)	7	M
2B8	P5	PS19 (tau P301S)	6	M
2B8	P1	WT (PS19 strain)	14	M
2B8	P2	WT (PS19 strain)	9	M
2B8	A11	M83 (α-syn A53T)	7	M
2B8	A12	M83 (α-syn A53T)	7	M
2B8	V23	M83 (α-syn A53T)	6	M
2B8	E36	M83 (α-syn A53T)	7	M
2B8	A25	M83 (α-syn A53T)	6	M
2B8	A20	M83 (α-syn A53T)	6	M

Table S6: List of the mice injected with labeled sdAb 2B8 (10 mg/kg).
The JNPL3 (tau P301L) and M83 (α-syn A53T) mice were homozygous for the transgene and the PS19 (tau P301S) were heterozygous for the transgene. The wild-type (WT) mice were of the same strain background as the JNPL3 and PS19 mice, respectively.

sdAb	Antigen	Phase	K_D	k_a	k_d
2D8	rec α-syn	Solid phase	248.9 ± 84.7 nM	2.84×10^4	7.04×10^{-3}
2D10	rec α-syn	Solid phase	95.4 ± 28.3 nM	8.31×10^4	6.09×10^{-3}
Poly-sdAb	rec α-syn	Solid phase	644.1 ± 200.1 nM	2.53×10^4	1.43×10^{-2}
2D8	P1	Solid phase	4.4 ± 2.2 nM	5.30×10^5	2.22×10^{-3}
2D10	P1	Solid phase	41.5 ± 2.5 nM	1.27×10^5	5.24×10^{-3}
Poly-sdAb	P1	Solid phase	78.0 ± 22.1 nM	4.45×10^4	3.36×10^{-3}
2D8	SP	Solid phase	18.2 ± 8.9 nM	1.10×10^5	1.57×10^{-3}
2D10	SP	Solid phase	4.4 ± 2.3 nM	4.94×10^4	2.75×10^{-4}
Poly-sdAb	SP	Solid phase	50.7 ± 6.9 nM	3.70×10^4	1.87×10^{-3}
2D8	rec α-syn	Solution phase	67.0 ± 18.5 nM	1.00×10^4	6.92×10^{-4}
2D10	rec α-syn	Solution phase	51.7 ± 15.9 nM	5.87×10^3	3.20×10^{-4}
Poly-sdAb	rec α-syn	Solution phase	144.1 ± 26.3 nM	6.91×10^3	1.04×10^{-3}
2D8	S1	Solution phase	84.1 ± 12.4 nM	2.68×10^4	2.34×10^{-3}
2D10	S1	Solution phase	46.7 ± 13.8 nM	1.96×10^4	9.89×10^{-4}
Poly-sdAb	S1	Solution phase	103.8 ± 11.9 nM	1.15×10^4	1.20×10^{-3}
2D8	LSS	Solution phase	86.1 ± 2.6 nM	8.01×10^3	6.89×10^{-4}
2D10	LSS	Solution phase	29.0 ± 4.7 nM	1.57×10^4	4.48×10^{-4}
Poly-sdAb	LSS	Solution phase	126.0 ± 22.9 nM	7.58×10^3	9.38×10^{-4}

Table S7: Binding, association, and disassociation constants of sdAb 2D8 and 2D10.
The sdAbs were reacted with recombinant human α-syn (rec α-syn), insoluble pellet fraction (P1) from human Lewy body dementia (LBD) brain, insoluble sarkosyl pellet (SP) fraction from Tg M83 mouse brain, soluble fraction (S1) from human LBD brain, and low speed supernatant (LSS) soluble fraction from Tg M83 mouse brain.

sdAb	Antigen	Phase	K_D	k_a	k_d
2B8	rec tau	Solution phase	14.2 ± 0.4 nM	3.31×10^{4}	4.67×10^{-4}
2B8	p-tau	Solution phase	32.9 ± 17.0 nM	1.11×10^{5}	3.01×10^{-3}
2B8	rec tau	Solid phase	49.3 ± 17.0 nM	2.00×10^{4}	9.54×10^{-4}
2B8	p-tau	Solid phase	78.3 ± 10.7 nM	5.66×10^{4}	4.24×10^{-3}
2B8	tau peptide 16	Solution phase	12.1 ± 6.6 nM	3.08×10^{4}	4.25×10^{-4}
2B8	tau peptide 18	Solution phase	5.2 ± 1.7 nM	1.84×10^{6}	9.74×10^{-3}

Table S8: Binding, association, and disassociation constants of sdAb 2B8. The sdAb was reacted with recombinant longest isoform (2N4R) of human tau (rec tau), recombinant 1N4R isoform hyperphosphorylated tau (p-tau), and epitope tau peptide 16 and 18.
